# LamNet: an alchemical-path-aware graph neural network to accelerate binding free energy calculations for drug discovery and beyond

**DOI:** 10.1093/nsr/nwaf559

**Published:** 2025-12-08

**Authors:** Renling Hu, Jialu Wu, Qun Su, Shimeng Li, Yang Li, Tianyue Wang, Yu Kang, Tong Zhu, Chang-yu Hsieh, Tingjun Hou

**Affiliations:** College of Pharmaceutical Sciences, Department of Pharmaceutical Sciences, Zhejiang University, Hangzhou 310058, China; Department of Scientific Intelligence, Shanghai Innovation Institute, Shanghai 200030, China; Algorithm Research and Development Department, CarbonSilicon AI Technology Co., Ltd, Hangzhou 310018, China; Zhejiang Provincial Key Laboratory for Intelligent Drug Discovery and Development, Institute of Pharmaceutical Sciences, Zhejiang University, Jinhua 321016, China; College of Pharmaceutical Sciences, Department of Pharmaceutical Sciences, Zhejiang University, Hangzhou 310058, China; Zhejiang Provincial Key Laboratory for Intelligent Drug Discovery and Development, Institute of Pharmaceutical Sciences, Zhejiang University, Jinhua 321016, China; College of Pharmaceutical Sciences, Department of Pharmaceutical Sciences, Zhejiang University, Hangzhou 310058, China; Zhejiang Provincial Key Laboratory for Intelligent Drug Discovery and Development, Institute of Pharmaceutical Sciences, Zhejiang University, Jinhua 321016, China; College of Pharmaceutical Sciences, Department of Pharmaceutical Sciences, Zhejiang University, Hangzhou 310058, China; Department of Scientific Intelligence, Shanghai Innovation Institute, Shanghai 200030, China; College of Pharmaceutical Sciences, Department of Pharmaceutical Sciences, Zhejiang University, Hangzhou 310058, China; Department of Scientific Intelligence, Shanghai Innovation Institute, Shanghai 200030, China; Department of Materials Science, Shanghai Academy of AI for Science, Shanghai 200030, China; College of Pharmaceutical Sciences, Department of Pharmaceutical Sciences, Zhejiang University, Hangzhou 310058, China; Zhejiang Provincial Key Laboratory for Intelligent Drug Discovery and Development, Institute of Pharmaceutical Sciences, Zhejiang University, Jinhua 321016, China; Department of Scientific Intelligence, Shanghai Innovation Institute, Shanghai 200030, China; College of Pharmaceutical Sciences, Department of Pharmaceutical Sciences, Zhejiang University, Hangzhou 310058, China; Zhejiang Provincial Key Laboratory for Intelligent Drug Discovery and Development, Institute of Pharmaceutical Sciences, Zhejiang University, Jinhua 321016, China; College of Pharmaceutical Sciences, Department of Pharmaceutical Sciences, Zhejiang University, Hangzhou 310058, China; Zhejiang Provincial Key Laboratory for Intelligent Drug Discovery and Development, Institute of Pharmaceutical Sciences, Zhejiang University, Jinhua 321016, China

**Keywords:** binding free energy, deep learning, graph neural network, thermodynamic transformation, LamNet

## Abstract

Accurate prediction of protein–ligand binding free energies is critical yet computationally demanding in drug discovery. Alchemical free energy methods (AFEMs) offer high accuracy but suffer from significant computational costs and complex modeling setup, such as tuning the λ-schedule of alchemical transformation. While conventional deep learning (DL) models may instantly predict binding affinity, they often require a large training set and exhibit limited generalizability across chemical space. To address these challenges, we introduce LamNet, an alchemical-path-aware graph neural network. LamNet integrates endpoint molecular states and the bridging alchemical path (parametrized by λ) into a physics-informed representation learning framework, explicitly modeling free energy changes along a chosen thermodynamic transformation pathway. Trained on molecular-dynamics-simulated data along alchemical pathways and incorporating data reliability metrics, LamNet accurately predicts relative binding free energies and absolute binding free energies, and optimizes λ-schedules to improve traditional AFEM convergence. Evaluations on diverse datasets (463 ligands, 16 proteins) demonstrate that LamNet achieves superior or comparable performance to state-of-the-art methods, including traditional AFEM, but with up to 1000-fold acceleration. These findings establish LamNet as a generalizable, physics-grounded, and cost-effective tool that not only accelerates computations but also provides a novel framework for integrating rigorous computational physics into modern DL-driven drug discovery workflows.

## Teaser

LamNet unifies AI and alchemical physics to overcome the accuracyefficiency tradeoff and generalization failure, establishing a universal modeling paradigm for scientific intelligence.

## INTRODUCTION

Free energy is a fundamental concept in statistical physics that is essential for understanding intricate molecular processes in biological systems and offers insights for rational drug design. Consequently, the development of efficient and accurate methods for free energy calculation is paramount in drug discovery [1,2]. For instance, in the context of lead optimization (LO), we demand highly precise assessment of binding free energy. An efficient and reliable relative binding free energy (RBFE) calculation method enables rapid determination of binding affinity differences between a lead compound and its variants, facilitating molecular structural modification [3]. On the other hand, a fast and accurate absolute binding free energy (ABFE) calculation method may provide a high-accuracy virtual screening [4], although such a reliable and computationally efficient ABFE method for large-scale virtual screening is still not yet available.

While molecular dynamic (MD)-based free energy methods (FEMs) serve as the predominant technique for obtaining high-accuracy RBFE and ABFE, they face a dilemma with the trade-offs between accuracy and efficiency. MD-based FEMs are broadly classified into two categories: physical pathway methods and alchemical free energy methods (AFEMs). The physical pathway approach, which simulates the ligand dissociation through a realistic physical pathway, is limited by the impractically long timescale required under current computational resources [5]. Amidst this difficulty, AFEMs, which rely on transforming one molecular state into another through a series of alchemical intermediates, offer a more reliable and feasible approach, demonstrating exceptional accuracy and a strong track record of successful applications in recent years [6–8]. While traditional AFEMs, such as free energy perturbation (FEP) [9] and thermodynamic integration (TI) [10], have proven to be very useful, there is still room for improvement. More recent approaches like λ-dynamics with bias-updated Gibbs sampling (LaDyBUGS) [11] and convergence-adaptive roundtrip method of combined-structure FEP (CS-FEP-CAR) [12] aim to further the balance between accuracy and expense. Nevertheless, this balance still remains suboptimal with existing methods, highlighting the urgent need for the development of novel methods.

On the other hand, in recent years, several studies have sought to integrate machine learning interatomic potentials (MLIPs) into AFEMs to address engineering challenges in MLIP implementation and further enhance computational accuracy. For instance, Zariquiey *et al*. developed a hybrid force field combining molecular mechanics (MM) with neural network potentials (NNPs) and implemented it within the alchemical transfer method (ATM) framework using the OpenMM simulation platform [13]. Similarly, Wang *et al*. integrated MM with diverse advanced MLIPs within the TI framework on the Amber simulation platform [14]. These approaches enable MLIPs to capture more precise and detailed protein–ligand interactions, thereby improving the accuracy of binding free energy calculations. However, this gain in accuracy comes at the cost of reduced computational efficiency.

Another considerable challenge of AFEM is that researchers tend to devote considerable time in parameterizing the λ settings or adapting the sampling strategy of λ, as the overlap of adjacent phase spaces is crucial for the convergence and accuracy of calculations, particularly in the case of large structural changes between the two end states. Non-optimal alchemical intermediates result in excessive or inadequate overlap between windows, leading to computational costs being inflated by up to ten times and large errors in free energy estimates. This hinders wider adoption of AFEMs in practical applications despite their supposedly highly accurate modeling capacity. In the face of this challenge, Zeng and Qian [15] and Zhang *et al*. [16] both utilized pre-run short-time simulations to assess the overlap of phase space. The major problem with such attempts is that pre-run simulations also entail significant computational expenses. Consequently, to maximize the full potential of AFEMs, it is desirable to devise protocols that automatically set up suitable windows at minimal cost while reducing manual trials and interventions.

Alongside AFEM, deep learning (DL)-based approaches have emerged in recent years as efficient alternatives for the large-scale prediction of protein–ligand binding affinities. These data-driven black box methods leverage neural architectures to achieve high-throughput predictions and have shown promising performance in both RBFE and ABFE tasks [17–19]. To improve model generalizability and interpretability, recent developments increasingly incorporate physics-informed priors [20–23]. However, current integration of physical knowledge remains limited to the level of structural or free energy formulation, lacking fundamental microscopic fidelity. Furthermore, existing models still rely heavily on static structural datasets, which are challenging to expand and thus constrain model generalization. Additionally, these models often fail to capture the entropy effects associated with the dynamical nature of protein–ligand interactions. Overcoming these challenges will require deeper integration of domain knowledge and the utilization of dynamic process-level data, such as those obtained from MD simulations, to enhance learning and data augmentation.

Herein, we present an alchemical-path-aware graph neural network (LamNet) to tackle the aforementioned problems: (1) classical AFEM demands significant computational resources; (2) tuning the set of λ parameters in AFEM requires expert knowledge and a substantial amount of trial and error; (3) data-driven scoring models exhibit limited generalizability and lack the consideration of dynamic transformation. LamNet is a graph neural network designed to predict cumulative free energies along a chosen alchemical pathway in AFEM, adhering closely to the principle of alchemical computational physics. To be specific, LamNet characterizes two endpoints and the λ parameters in AFEMs, whereas its representation learning phase describes how ligands interact with protein pockets through alchemical transformations and determines different pathways within the thermodynamic cycle. In addition, the training data that models the transformation of alchemy comprises the process data generated by ATM (one of the newly proposed AFEMs) [24], with the accuracy of each data point being taken into account during the training process. LamNet can accomplish three applications for different types of training data and downstream algorithms: (1) reliable and efficient prediction of RBFE; (2) efficient and scalable prediction of ABFE; (3) prediction and optimization of λ settings for AFEM with improved convergence. It is worth emphasizing that LamNet is essentially a DL framework that can be integrated with AFEMs other than ATM. In this work, ATM is chosen mainly for its theoretical simplicity and ease of large-scale simulations for training data generation. LamNet can also be built upon the more traditional FEP approach, but we need to re-compute the training dataset according to the standard alchemical transformation paths of FEP. More discussion about this is provided in Supplementary Information. Hence, we only illustrate how LamNet works with ATM.

LamNet has been evaluated over 463 ligands across 16 proteins for RBFE prediction, achieving RMSE of 1.21 kcal/mol and Pearson *r* of 0.61 on 8 targets, 1.30 kcal/mol and 0.63 on the remaining 8 targets, demonstrating performance that is comparable to, and in some cases superior to, various mainstream and novel methods or models. Furthermore, through few-shot learning (where a limited amount of data is available), LamNet can attain results that surpass FEP (Pearson *r* 0.67 vs. 0.65), while reaching a speed boost—completing predictions in <1 GPU hour compared to ∼4000 GPU hours required by FEP. Additionally, compared with other data-driven models, LamNet exhibits higher data efficiency and greater application potential, which has been further validated in our activity cliff (AC) challenge. These results highlight LamNet’s potential as a generalizable, cost-effective, and physically grounded method for predicting free energies in real drug development scenarios. For ABFE prediction, LamNet has been assessed over 4 guests on 1 host and provided reasonable predictions with limited training, exhibiting promise to transfer to biomolecular systems. Furthermore, for challenging cases, LamNet provides a robust alternative to accelerate convergence (e.g. 8 h vs. 52 h and 3.5 h vs. 16 h) while maintaining reliability through the optimization of λ parameters, which is illustrated via CDK2_1oiu_1h1q and MCL1_32_46 case studies.

## RESULTS

### Model architecture

**Figure 1.**
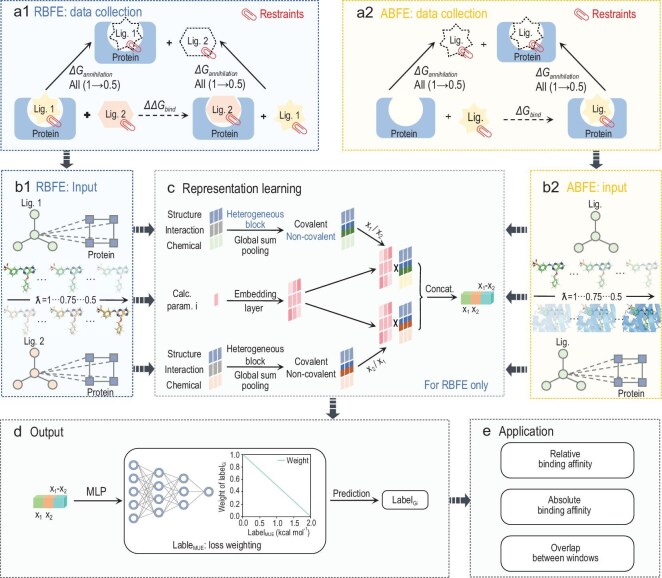
The workflow of LamNet. (a) The data collection phase collects the cumulative free energy across each λ window in the AFEM ((a1) RBFE calculations, (a2) ABFE calculations), represented here as ATM. (b) The input phase ((b1) RBFE data, (b2) ABFE data) consists of two endpoints and λ-parameterized alchemical intermediates in the AFEM. (c) The physics-informed representation learning derives complex-pair (RBFE) or complex-ligand-pair (ABFE) representations for each λ window along different pathways within the thermodynamic cycle. The heterogeneous block is only used for complex-type graphs rather than ligand-type graphs. (d) In the output phase, window-level free energies are predicted using a MLP. (e) A multi-objective application phase aimed at expediting RBFE and ABFE calculations via direct prediction or indirect convergence optimization.

LamNet is an alchemical-path-aware graph neural network designed to significantly accelerate and preserve the accuracy of (relative and absolute) binding free energy calculations for biomolecular systems. As illustrated in Fig. 1, the model’s workflow is structured into three main stages: input phase, representation learning phase and output phase.

Initially, in the input phase, LamNet prepares distinct inputs for RBFE (protein–ligand complexes) and ABFE (ligand and protein–ligand complex). It not only takes in two endpoint molecular states but also λ parameters, which implicitly encode a series of desired alchemical intermediates. During the representation learning stage, LamNet first extracts node-level, atomic features from molecular structures and embeds λ parameters. These features are then used by a message-passing neural network to determine covalent and non-covalent interactions among atoms in the endpoint molecular states. Once the chemical connectivity and the inter-atomic potential is settled, LamNet conducts graph-level representation learning for each λ-parameterized alchemical intermediate from different pathways within the thermodynamic cycle. Next, LamNet employs a well-trained multilayer perceptron (MLP) to predict window-level free energies along the λ-parameterized alchemical path at the output phase. Finally, RBFE or ABFE can be simply obtained by the predicted cumulative energies from different pathways defined in AFEMs, and λ settings can be attained by predicting and controlling the energy difference between adjacent windows to not exceed a specific threshold (here, 10 kcal/mol). Further algorithmic details about each stage of the workflow and derivation of invariance can be found in Supplementary Information.

LamNet is essentially a novel framework that combines black-box DL models with traditional computational physics, and it can be combined with any AFEM, such as FEP. In this work, we chose to train LamNet, based on ATM-generated data, for three main reasons: first, the theoretical simplicity of ATM; second, the computational convenience of ATM, which facilitates the access of large-scale training data; and third, there have already been several successful studies that combined ATM with DL force fields [13,25]. To train LamNet with another underlying AFEM, one needs to recalculate the training set along the corresponding alchemical path of a new method. More discussion about this is provided in Supplementary Information. Given the computational cost, we only illustrate the novel concept and outstanding results of LamNet with ATM.

LamNet’s utilization of process-level computational data holds significant implications for both the artificial intelligence (AI) and computational chemistry communities. From the AI perspective, LamNet breaks through the conventional limitation of one-to-one ‘static structure–experimental affinity’ data, opening a new avenue to overcome bottlenecks in data augmentation. From the perspective of computational chemistry, LamNet provides an innovative strategy for transforming otherwise discarded simulation process data into valuable training resources, thereby enhancing the scientific values of molecular simulations and fostering a more collaborative environment for data sharing.

Learning how the free energy of a molecular system more smoothly changes over the alchemical path endows LamNet an unparalleled generalizability and reliability as a data-driven method. In contrast to conventional DL models that treat the prediction of free energy as a single-structure, black-box regression problem, LamNet closely follows the AFEM formulation and uniquely and explicitly models the free energy change of the molecular system along the alchemical path (as shown in Fig. 1b1, 1b2, and 1c). To be more precise, LamNet models interaction dynamics consistent with AFEM principles by performing multiplicative fusion between complex interaction graphs and parameter representation graphs. Besides, LamNet encodes both legs of a typical thermodynamic cycle in AFEMs, as illustrated in Fig. 1a1 and 1a2, through the order of feature concatenation, enabling the model to distinguish free energy contributions from different transformation pathways. Moreover, pathway-dependent cumulative training (with λ-parametrized alchemical free energies estimated from MD simulations) enables LamNet to comprehend the slow and dynamical transition of free energy landscapes while also improving its capacity to identify fundamental physical trends that dictate alchemical transformations. To enhance robustness and reliability, LamNet adopts a loss weighting strategy that accounts for the computational accuracy of the training data, prioritizing high-precision samples while reducing the influence of noisy data.

### Accuracy and generalization performance of LamNet

To investigate the accuracy and generalizability of LamNet in predicting λ-parameterized, window-level alchemical free energies, we conducted a comprehensive evaluation on 12 targets: BACE, CDK2, JNK1, MCL1, p38, thrombin, TYK2, PTP1B, hif2a, pfkfb3, syk, and tnks2, which also comprise our dataset (see Methods and Table S1 for details), involving both target-specific testing and leave-one-target-out testing experiments.

**Figure 2.**
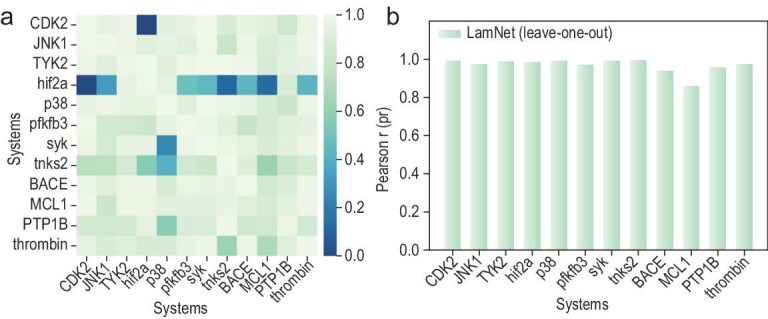
Generalization performance of LamNet in the λ-parameterized, window-level free energy predictions. (a) Heatmap of target-specific learning, where LamNet is trained on a single target and tested in a zero-shot manner on the other 11 targets. Green regions indicate strong generalization capability, while blue regions represent cases where minimal fine-tuning is beneficial. The corresponding Pearson *r* values are labeled within the heatmap. (b) Leave-one-target-out validation, where LamNet is trained on 11 targets and tested on the excluded target. High Pearson *r* values across all systems demonstrate LamNet’s robust generalization ability in predictions, effectively learning transferable molecular interactions and fundamental alchemical free energy principles across diverse targets.

The left panel of Fig. 2 illustrates that, when trained on the alchemical MD-simulated data for a single target, LamNet is capable of predicting cumulative free energies over various windows on the validation set of unseen targets under a zero-shot scenario (Pearson *r* >0.85 in the majority of cases). Furthermore, the right panel of Fig. 2 illustrates that LamNet, when trained on 11 targets and evaluated on the validation set of the remaining target, consistently exhibits strong generalization performance (Pearson *r* >0.85 across all targets).

These outcomes certainly imply that LamNet may shine under a variety of out-of-distribution (OOD) situations that conventional data-driven methods tend to fail or struggle with. To be specific, due to its inherent integration with the underlying physics, LamNet may have a better understanding of dynamic interactions and physical trends in OOD scenarios, which is currently a bottleneck of black-box models. Moreover, LamNet utilizes a different data distribution compared to conventional DL models, and the dynamic window-level energy distributions of alchemical intermediates, particularly RBFE for various targets, might show relatively little variation. Furthermore, the process modeling leads to a smoother feature space, which also provides an advantage for LamNet’s generalization. We came to the same conclusion in terms of accuracy **(**root mean square error (RMSE)). Detailed data of both experiments are provided in the source data of Fig. 2 (see Supplementary Information). In addition, we conducted similarity analyses on the pocket for every target using BLASTP (sequence similarity) [26] and ProBiS (structural similarity) [27], and results demonstrate the absence of similarity in the binding regions across these 12 targets, hence further substantiating the above conclusions. The specific results of BLASTP and ProBiS are provided in the source data of Fig. 2.

### Relative binding free energy prediction: LaDyBUGS benchmarking

Next, we analyzed the performance of LamNet for the most common free-energy calculation task in drug design: RBFE prediction. For this investigation, we considered three test systems used in the study of the LaDyBUGS method, reported by Robo *et al*. [11]. LaDyBUGS, one of the fastest AFEMs available improved through fundamental theory, treats λ as a dynamic variable with a fictitious mass and adjusts it by an aggressive dynamic bias. To gain a better understanding of LamNet’s performance, we compared LamNet against LaDyBUGS and one of the most classical AFEMs termed TI. The three systems were cMet (10) [28], thrombin (11) [29], and pfkfb3 (10) [30], with the figures in parentheses representing the number of ligands involved with each target. Perturbations among ligands were carried out via star mapping, as illustrated in Fig. S4b, ensuring complete connection and comprising only charge-neutral perturbations. The ligand having the smallest perturbed structure, derived from the maximum substructure, was chosen as the starting point (the reference ligand). Δ*G* was calculated with all experimental values and the ΔΔ*G* of ligands as detailed in Supplementary Information. To further illustrate the generalization capability of LamNet and its practical applicability in drug design, LamNet’s predictions for all targets were obtained by considering each target as an external test set, in which a leave-one-target-out learning strategy was used once the target duplicated with targets in our build-up dataset.

Symmetric perturbations (ΔΔ*G* = 0.00 kcal/mol) were performed on the reference ligands for each system, and all results approximate 0.00 kcal/mol, with errors around on the order of 1e^−5 ^kcal/mol, revealing that LamNet truly learned the underlying physical trend of structural perturbations.

**Figure 3.**
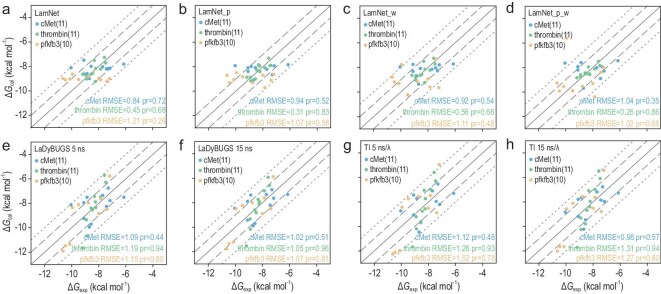
Overall RBFE calculation results on the LaDyBUGS case study. Correlation between the calculated and experimental Δ*G* for 3 protein-ligand systems over 8 methods (a–h), with the names of methods on the top left of the panels, the RMSE and Pearson *r* of each system on the bottom right. Dots on the solid line indicate that the calculated values are completely correlated with the experimental values, dots within the area of the dashed line indicate that the error is <1.0 kcal/mol, and dots within the area of the dotted line indicate that the error is <2.0 kcal/mol. Panels (a–h) correspond to LamNet, LamNet_p, LamNet_w, LamNet_p_w, LaDyBUGS (5 ns), LaDyBUGS (15 ns), TI (5 ns/λ), and TI (15 ns/λ), respectively.

In comparison to LaDyBUGS and TI, LamNet and its variants exhibit increased quantitative accuracy along with comparable consistency in predicting RBFE. Readers can find more details of LamNet and its variants in Supplementary Information. Results are shown in Fig. 3, where subplots (a–d) correspond to LamNet and its variants (LamNet, LamNet_p, LamNet_w, LamNet_p_w), and subplots (e–h) show results for LaDyBUGS and TI under different sampling settings. Unless stated otherwise, the default configuration of LamNet mentioned in the text is the variant with a loss weighting strategy (LamNet_w). In the cMet system, LamNet delivers superior performance in both accuracy and ranking, with an RMSE of 0.84 kcal/mol and a Pearson *r* of 0.72. In the thrombin system, LamNet_p_w exhibits higher accuracy, with an RMSE of 0.31 kcal/mol, even though its ranking capability is slightly lower, with a Pearson *r* of 0.86. The conclusion for the pfkfb3 system is analogous to that of thrombin, with an RMSE of 1.02 kcal/mol and a Pearson *r* of 0.68. More detailed data can be found in Table S2. Detailed statistics are available in the source data of Fig. 3.

Furthermore, LamNet dramatically elevates computational efficiency due to its fundamentally data-driven approach. To elaborate further, TI necessitates ∼5 ns per window for a pairwise perturbation, estimated at ∼2000 GPU hours per target, whereas LaDyBUGS applies 5 ns of total sampling, estimated at ∼20 GPU hours per target. By contrast, LamNet completes its predictions within 1 minute per target on a single GPU, achieving a substantial speedup of over three orders of magnitude. This considerable efficiency enhancement is accomplished without compromising accuracy, rendering LamNet especially valuable for LO.

### Relative binding free energy prediction: FEP1 and FEP2 benchmarking

Following the encouraging outcomes noted on the LaDyBUGS benchmark, we further evaluated the performance of LamNet on two large-scale datasets (FEP1 and FEP2) consisting of chemically and structurally diverse protein–ligand complexes, which had already been extensively used for benchmarking studies of FEP and similar free energy calculation methods [6,7]. The main workflow and concerns remained in line with those previously stated. Also, identical results were attained in the symmetric perturbation experiments.

**Figure 4.**
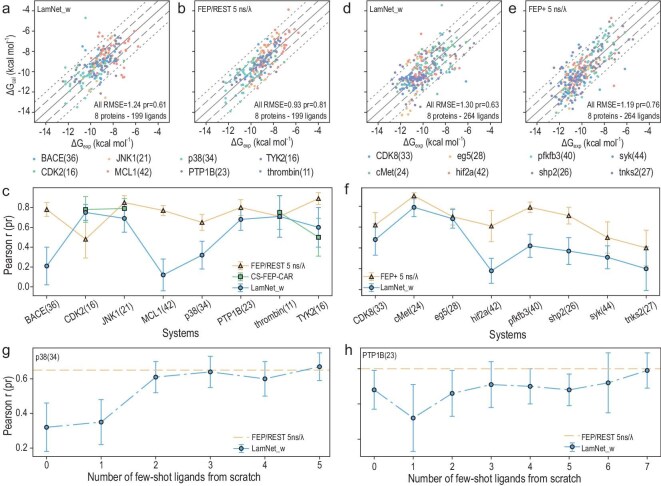
Overall RBFE calculation results on the FEP1 and FEP2 sets. (a) Correlation between the LamNet-calculated and experimental Δ*G* on the FEP1 set. (b) Correlation between the FEP/REST (5 ns/λ)-calculated and experimental Δ*G* on the FEP1 set. (c) Line plot of the Pearson *r* of 3 methods on the FEP1 set. Blue represents LamNet, yellow represents FEP/REST (5 ns/λ), and green represents CS-FEP-CAR. CS-FEP-CAR is only tested on 4 targets. (d) Correlation between the LamNet-calculated and experimental Δ*G* on the FEP2 set. (e) Correlation between the FEP+ (5 ns/λ)-calculated and experimental Δ*G* on the FEP2 set. Dots on the solid line indicate that the calculated values are completely correlated with the experimental values, dots within the area of the dashed line indicate that the error is <1.0 kcal/mol, and dots within the area of the dotted line indicate that the error is <2.0 kcal/mol. (f) Line plot of the Pearson *r* of 2 methods on the FEP2 set. Blue represents LamNet and yellow represents FEP+ (5 ns/λ). Error bars represent the standard deviation of the Pearson *r* performance, obtained by Bootstrapping. (g) Improvement in the Pearson *r* performance of LamNet on the p38 system as the number of few-shot ligands increases. (h) Improvement in the Pearson *r* performance of LamNet on the PTP1B system as the number of few-shot ligands increases. Yellow dashed line represents the performance of FEP/REST (5 ns/λ).

Figure 4a**–**c illustrate that the FEP1 dataset has 8 protein targets, including BACE (36) [31], CDK2 (16) [32], JNK1 (21) [33], MCL1 (42) [34], p38 (34) [35], PTP1B (23) [36], thrombin (11) [29], and TYK2 (16) [37,38], and a total of 199 ligands. The numbers in parentheses indicate the number of available ligands for each target, utilized to differentiate between identical targets present in the prior benchmark (the LaDyBUGS benchmark). It should be noted that thrombin (11) in the LaDyBUGS benchmark and FEP1 dataset are identical. For this benchmark, LamNet achieves a competitive overall RMSE of 1.21 kcal/mol and a Pearson *r* of 0.61. Further, for the CDK2 and thrombin systems, LamNet manifests a comparable and even superior Pearson *r* (0.75 and 0.71, respectively) when compared to classical FEP/REST (5 ns/λ; 0.48 and 0.71, respectively), reflecting better concordance with the results of experiments. Additionally, LamNet demonstrates highly competitive results when compared to CS-FEP-CAR on four systems: CDK2, JNK1, thrombin, and TYK2. More details can be found in Table S3.

As shown in Fig. 4d–f, the FEP2 dataset contains 264 ligands toward 8 protein targets: CDK8 (33) [39], cMet (24) [28], eg5 (28) [40], hif2a (42) [41], pfkfb3 (40) [30], shp2 (26) [42,43], syk (44) [44], and tnks2 (27) [45]. The numbers in parentheses indicate the number of ligands available for each target in this dataset, utilized to differentiate between identical targets present in the prior benchmark (the LaDyBUGS benchmark). In this benchmark, LamNet achieves a notable overall RMSE of 1.30 kcal/mol and a Pearson *r* of 0.63. Moreover, while LamNet exhibits slightly lower performance than FEP+ across all targets, the general trend of its curve is consistently compatible with that of FEP+ . More detailed data can be found in Table S4. Detailed statistics are located in the source data of Fig. 4.

Interestingly, LamNet favors learning more flexible binding modes with superior generalizability in comparison to conventional data-driven approaches, benefiting from its ability to learn dynamic interaction features from alchemical pathways. As shown in Fig. S1a, the hif2a system exhibits a more rigid pocket with lower root mean square fluctuation (RMSF) values, which correlates with LamNet’s reduced performance in target-specific zero-shot learning. Similar trends are observed in pfkfb3, BACE, and MCL1 systems (Fig. S1b and d), all featuring limited flexibility and deeper potential wells. These rigid targets coincide with higher RMSE outliers in the LaDyBUGS benchmark, particularly where ligand pairs show large experimental RBFE differences (Fig. 3c and d). Furthermore, both the CDK2 and thrombin systems achieve superior performance over FEP on the FEP1 benchmark (Fig. 4c), which can be attributed to their comparable flexible binding pockets (Fig. S2). On the other hand, the training data derived from short-time (1 ns per λ-window) simulations may lack dynamic information related to escaping from the deep potential well or crossing the high energy barrier. Nevertheless, we anticipate this relative shortcoming of LamNet can be addressed by running longer MD simulations or exploiting enhanced sampling techniques for various targets and binding modes. Readers can find more analysis on this topic in Supplementary Information.

The aforementioned findings on the FEP1 and FEP2 datasets demonstrate LamNet’s outstanding predictive and generalizable ability. Beyond that, LamNet presents an invaluable improvement in efficiency as a data-driven model, as previously stated. Our comparisons indicate that traditional AFEMs, such as FEP+, often necessitates ∼5 ns of sampling per λ window, resulting in an overall expense of ∼4000 GPU hours per target. The newly stated CS-FEP-CAR approach enhances efficiency to a degree but still requires ∼500 GPU hours to carry out all pairwise perturbations within a network. In contrast, LamNet makes predictions for a whole set in <1 minute on a single GPU, yielding a three-to-four orders of magnitude of speedup.

Other results further emphasize LamNet’s noteworthy potential as a scalable and reliable RBFE prediction method. As illustrated in Fig. 4g and h, LamNet exhibits remarkable few-shot learning proficiency: when given just a few labelled data of ligand pairs and trained from scratch, the model can even surpass traditional FEP approaches in ranking accuracy. This indicates that while LamNet’s zero-shot performance on certain targets may be less accurate than the traditional FEP, it is capable of rapidly adapting and providing more accurate results when limited labeled data are available. It is especially valuable in the early stages of drug discovery when experimental data is scarce.

We also evaluated the performance of LamNet and several mainstream scoring models with greater focus on the 16 targets of FEP1 and FEP2. No single method consistently outperforms the others; rather, each achieves top performance on some targets and poor performance on others, highlighting their varying effectiveness across different scenarios. It should be noted that these scoring models were trained on the PDBbind-like dataset, which includes targets such as cMet, thrombin, pfkfb3, and so on. Given that LamNet completed all predictions in a strict leave-one-target-out setting, the comparison may even underestimate its generalization capability. Therefore, the strong performance of LamNet in this stricter pattern highlights its robustness and practical applicability in drug design. Detailed data for the comparison can be found in Fig. S3, and Tables S5 and S6. A point to note is the comparison with PBCNet, as it is the only baseline model that is strictly data-driven, i.e. it directly predicts RBFE. According to our analysis, it seems that LamNet holds two advantages over PBCNet. First, LamNet is trained on roughly 6000 data points, which is about one hundredth of that of PBCNet (600k). While traditional data-driven models typically require large amounts of data to learn end-to-end relationships, LamNet achieves comparable accuracy and superior generalization performance with just 1% of the amount of data. Second, given the limited quality of the training data and the opportunity to obtain further computational process data, LamNet demonstrates significantly more potential than traditional models. On the one hand, current training data of LamNet is derived from short-time (1 ns per λ-window) simulations, and the quality of the training data somewhat limits the performance of LamNet. On the other hand, traditional DL models have reached a bottleneck in the utilization of experimental data. However, the computational process data is more easily obtainable in comparison to the experimental data. Hence, the performance of LamNet can be readily enhanced by improving the quality or quantity of training data. Readers can find more detailed discussion on the impact of data quality on LamNet’s performance in Supplementary Information.

In addition to the aforementioned scoring models, structure-prediction-based models such as Boltz2 have also demonstrated strong performance in free energy prediction tasks [46]. Here, we provided a brief comparison between LamNet and Boltz2. On four FEP1 targets (CDK2, TYK2, JNK1, and p38), the overall Pearson correlation coefficients achieved by LamNet and Boltz2 are comparable (0.59 vs. 0.66, respectively). Boltz2 integrates a free energy prediction module into a structure-prediction framework, and its high accuracy can be attributed to its flexibility in handling initial structural inputs. However, its generalization capability is extremely limited. Although Boltz2 can predict binding free energies even in the absence of 3D structures, this limitation significantly reduces its practical utility. Furthermore, the inclusion of a structure-prediction step in Boltz2 introduces extra computational cost, reducing its overall efficiency.

To summarize, these results highlight LamNet’s strong adaptability, data efficiency, and physically grounded generalization capability, underscoring its potential as a reliable and scalable RBFE prediction framework for real-world drug discovery applications.

### Relative binding free energy prediction: activity cliff challenge

To further validate the strong generalization capability of LamNet and its potential utility in real-world drug discovery scenarios, we evaluated LamNet and several baseline models on AC scenarios. Specifically, we employed the BindingNet-AC benchmark, which contains 51 131 ligand pairs that meet the classical AC criteria along with well-constructed 3D complex structures [47]. Given that this benchmark requires accurate prediction of relative binding affinity differences between ligand pairs, models with ambiguous physical interpretability (e.g. RTMScore and GenScore) were excluded. After removing a small number of structures with invalid valence, a total of 51 022 ligand pairs were used for RBFE prediction. It is worth noting that LamNet was trained on the full 12-target dataset here, eliminating the underestimation of LamNet mentioned earlier. The results are summarized in Table 1.

**Table 1.** Comparative performance of RBFE prediction on the BindingNet-AC benchmark.

		Models				
Metrics		LamNet	PBCNet	PBCNet2	PIGNet2	OnionNet2
ddG^a^	MUE^b^	2.35 ± 0.01	2.38 ± 0.00	2.61 ± 0.01	2.21 ± 0.01	**2.13 ± 0.00**
	RMSE^c^	2.88 ± 0.01	2.62 ± 0.01	2.98 ± 0.01	2.56 ± 0.01	**2.35 ± 0.01**
	pr^d^	**0.18 ± 0.00** ^f^	−0.20 ± 0.00	−0.31 ± 0.00	0.11 ± 0.00	**0.18 ± 0.00**
	τ^e^	**0.11 ± 0.00**	−0.14 ± 0.00	−0.20 ± 0.00	0.08 ± 0.00	**0.11 ± 0.00**

a All metrics are reported from the perspective of RBFE (ddG). ^b^Mean unsigned error in kcal/mol. ^c^Root mean square error in kcal/mol. ^d^Pearson *r*. ^e^Kendall *r*. ^f^Best-performing results for each metric are highlighted in **bold**.

As shown, most existing models exhibit a dramatic decline in performance under AC scenarios, with Pearson *r* typically below 0.2, indicating that OOD generalization remains a major challenge for data-driven approaches. Interestingly, the top-performing models, LamNet and OnionNet2, are those that incorporate physical knowledge, suggesting that physics-informed modeling plays a crucial role in mitigating generalization failures. Moreover, LamNet achieves strong performance despite being trained on a relatively small and restricted dataset, highlighting the robustness and scalability of its architecture. These findings reinforce LamNet’s potential as a powerful and generalizable RBFE prediction framework for real-world drug discovery applications. Detailed statistics are provided in the source data of Table 1 (see Supplementary Information).

### Absolute binding free energy prediction: cucurbit[7]uril benchmarking

**Figure 5.**
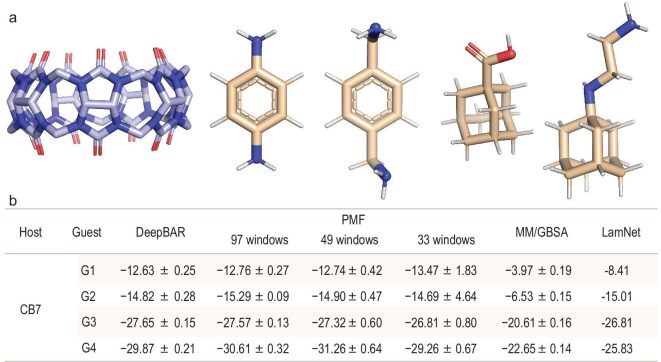
The results of ABFE calculations. (a) The cucurbit[7]uril (CB7) host-guest system. (b) Comparison of ABFE of the 4 guests calculated by DeepBAR, PMF, MM/GBSA, and LamNet. The unit of ABFE is kcal/mol.

Next, we considered another mainstream application scenario of AFEM in drug discovery: the ABFE calculations. In light of the intractable computational expense associated with the preparation of ABFE datasets on biomolecular system, we chose to present LamNet results in the SAMPL Challenge’s host-guest systems. The host-guest system incorporates significant impediments in protein–ligand binding free energy calculations, encompassing factors such as conformation, protonation, and related aspects, which is pivotal for propelling methodological advancements and facilitating the identification of novel drug candidates [48–53]. To be specific, we evaluated LamNet’s performance on the cucurbit[7]uril (CB7) host-guest system (Fig. 5a), as used in the innovative work of DeepBAR, published by Ding and Zhang [54]. DeepBAR represents one of the most exemplary attempts to combine DL with conventional ABFE calculations. To gain a better understanding of LamNet’s performance, we compared LamNet with DeepBAR, the potential of mean force (PMF) method [55,56], and the molecular mechanics generalized Born and surface area continuum solvation (MM/GBSA) [57]. Given the scarcity of training data for ABFE and the fact that weighting would further aggravate data sparsity, we trained LamNet without a loss weighting strategy in the subsequent ABFE-related experiments. For clarity, they will hereafter still be referred to as LamNet.

The results of LamNet’s predictions on the CB7 system are summarized in Fig. 5b. It can be seen that LamNet achieves a comparably correct ordering of ABFE for the four molecules, despite its predictions being slightly less accurate than those of DeepBAR and PMF. In comparison to MM/GBSA, which relies on empirical formulas, LamNet yields more accurate results. Specifically, for guest G3, LamNet’s predicted Δ*G* of −26.81 kcal/mol roughly aligns with DeepBAR (−27.65 kcal/mol) and PMF (−27.32 kcal/mol), while MM/GBSA significantly underestimates the binding affinity at −20.61 kcal/mol. The results confirm that LamNet is capable of offering reasonable predictions with limited training data.

Nevertheless, due to computational resource constraints, LamNet was trained with rather noisy and few data points. Its current performance was actually encouraging. To elaborate, LamNet is trained on a dataset comprising over 600 data points derived from short-time (1 ns per λ-window) simulations, while DeepBAR is trained on a dataset of 1600k data points accumulated from long (20 ns and 100 ns) trajectories. Consequently, if the data quality and quantity could be improved, we posit that LamNet may possibly surpass DeepBAR or even PMF to become a powerful tool that combines high accuracy and high efficiency in the future. Furthermore, given the strong generalization performance previously demonstrated by LamNet, it is anticipated that it will also be capable of transferring to biomolecular systems with minimal difficulty, a feat that is nearly unattainable for DeepBAR and traditional computational methods.

### λ parameter optimization: CDK2_1oiu_1h1q and MCL1_32_46 case study

In contrast to the direct prediction of free energy, LamNet can additionally improve the calculational efficiency of AFEM by helping to assess the overlap between adjacent windows using window-level free energy predictions and optimize λ window settings. This is the third possible application scenario that we have explored with LamNet in this study. Drawing from the research of Zeng and Qian [15], we utilized the free energy difference between neighboring windows to assess their overlap, with a threshold established at 10 kcal/mol. The free energy for each window is estimated at intervals of 0.001 throughout the range of 0 to 1, and the expected window setups are thereafter provided with an optimization algorithm. To figure out if this improves the efficiency of standard AFEM calculations, we studied the convergence of calculations before and after the optimization on two complexes (CDK2_1oiu_1h1q and MCL1_32_46). Simulations were performed using AToM-OpenMM, as per Gallicchio *et al*. [58]. The estimated ΔΔ*G* and their corresponding standard deviations were obtained by UWHAM (bin-less weighted histogram analysis method) [59].

**Figure 6.**
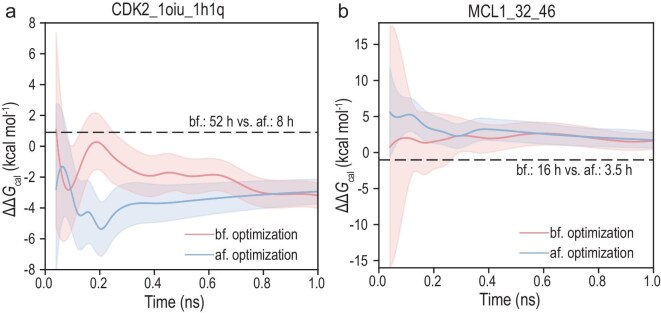
The results of optimized classical RBFE calculations. Left and right panels represent the comparison of convergence curves for ligand pairs CDK2_1oiu_1h1q (a) and MCL1_32_46 (b), respectively. The pink line denotes calculations performed across windows of 22 uniformly spaced windows ranging from 0 to 1, whereas the blue solid line indicates computations performed using the windows provided by LamNet_w and the posterior optimization algorithm. The color shading corresponds to the standard deviation. A comparison of the computation time with and without optimized λ settings is presented in the middle right of the panel.

As illustrated in Fig. 6, the numerical convergence of the algorithm has been noticeably improved by LamNet-optimized λ settings. The overall calculation time for CDK2 decreased from 52 h to 8 h, resulting in an ∼6.5-fold acceleration along with smaller uncertainty. Similar to that, for MCL1, the computational time shortened from 16 h to 3.5 h, accompanied by decreased variance in free energy calculation. These results demonstrate that LamNet’s prior assessment of window-level free energies offers insightful instructions on how to reduce unnecessary λ windows in relatively flat regions in the free energy landscape and setting more windows in areas of nonlinear transformation, thus optimizing λ window settings (see source data of Fig. 6).

In contrast to traditional trial-and-error λ tuning or fixed-spacing schemes, LamNet’s prediction-driven adaptive window settings do not depend on prior simulations or the empirical expertise of computational scientists. These results imply that LamNet could serve as a valuable module to greatly ease the automation setup of an AFEM calculation. When applied to various perturbations, such integration may result in significant decreases in overall computational costs of RBFE studies. Furthermore, this also emerges as a compelling alternative for some systems that find data-driven models challenging.

## DISCUSSION

We introduce LamNet, a physics-informed and alchemical-path-aware graph neural network, as our attempt to formulate a comprehensive and transformative solution to many persistent issues in alchemical free energy calculations. LamNet integrates physical principles with alchemical MD-simulated data, bridging the gap between mechanistic modeling and data-driven prediction. It facilitates reliable RBFE and ABFE predictions across a diverse chemical space while also facilitating the optimization of λ schedule for a typical AFEM, thereby improving the overall efficiency of a traditional method.

In the process of building LamNet, we have identified a new sweet spot for combining computational physics and DL. Compared to any end-to-end, black-box method for predicting the binding affinity of biomolecules, LamNet stands out due to its reliability and unparalleled generalizability (as it follows an alchemical path to make a series of easier problems). On the other hand, when compared to any traditional AFEM, LamNet easily offers a three-order-of-magnitude speedup (as it is inherently a data-driven predictor). Its outstanding performance across multiple benchmarks illustrates its capacity to substantially decrease computing expenses while preserving or even enhancing prediction accuracy.

LamNet demonstrates a new way to leverage DL to scale up AFEM calculations for real-world drug discovery. In the future, we will persist in optimizing and acquiring high-quality process data for LamNet training, as well as refining LamNet’s internal strategies for tackling different chemical systems, with the ultimate objective of creating a robust, physics-grounded AI framework for drug development.

## METHODS

### Datasets

We established two RBFE datasets using the ATM: ATM_MM (classical MM) and ATM_MM/NNP (hybrid ML/MM with ANI-2x for the intra-ligand region) [60]. Both include 8 pharmaceutically relevant targets [13,58] and were simulated with ff14SB [61], TIP3P water, GAFF2 [62], and AM1-BCC charges. A total of 607 ligand pairs were computed and low-quality or failed perturbations were removed, leaving 307 valid pairings (6754 points) and 5478 training samples. ABFE datasets were generated for 15 SAMPL host-guest systems representing various pocket chemotypes [48–53]. A total of 134 host-guest pairs were simulated using ATM with GAFF2, TIP3P water, and AM1-BCC charges. After quality screening, 38 pairings (682 training points) were retained. ATM simulations were performed on NVIDIA A100 GPUs and H100 GPUs. Full details of the simulation, screening strategy, and model input processing are provided in Supplementary Information.

### Trainings

LamNet predicts window-level free energies and is trained with a weighted MSE loss that emphasizes more reliable samples. We use AdamW [63], linear warm-up, ReduceLROnPlateau [64], weight decay, and early stopping. Multi-target training encourages generalization, while single-target training prioritizes convergence stability. Performance is evaluated using RMSE and Pearson *r*. Full hyperparameter settings, weighting strategy, and ablation experiments are given in Supplementary Information.

### Benchmarks

LamNet was thoroughly tested on a variety of benchmarks, including LaDyBUGS [11], FEP1/FEP2 [6,7], BindingNet-AC [47], and DeepBAR [54]. These datasets include large perturbations, charge changes, ring breakage, scaffold hopping, activity cliffs, and host-guest binding scenarios, all of which prove LamNet’s transferability and robustness across chemical and biological spaces. Further details about the benchmark, experimental setup, and specific discussions are provided in Supplementary Information.

## Supplementary Material

nwaf559_Supplemental_Files

## Data Availability

The dataset used to validate and test LamNet is available in the GitHub repository: https://github.com/RenlingHu/LamNet. We also provide part of our training data (2 targets for RBFE and 2 hosts for ABFE) to help readers understand the network architecture and training process of LamNet. The source data for Figs 3, 4, and Table 1, including the final calculated ΔΔ*G* and Δ*G* for all the ligands, are given in the attached Excel file for download. The source data for Fig. 6, including the estimated ΔΔ*G* and standard deviation, are also supplied.

## CODE AVAILABILITY

The source code of LamNet is available in the GitHub repository: https://github.com/RenlingHu/LamNet
