# Supplementary material for: LamNet: an alchemical-path-aware graph neural network to accelerate binding free energy calculations for drug discovery and beyond
Source: Natl Sci Rev. 2025 Dec 8;13(3):nwaf559. doi: 10.1093/nsr/nwaf559 (PMC12887304; doi:10.1093/nsr/nwaf559)
Supplement: nwaf559_Supplemental_Files [file nwaf559_supplemental_files.zip › 1436-SI-updated.pdf]

Supplementary Information

LamNet: an Alchemical-Path-Aware Graph Neural Network to Accelerate Binding Free Energy Calculations for Drug Discovery and Beyond

Renling Hu[1,2,3,4], Jialu Wu[1,4], Qun Su[1,4], Shimeng Li[1], Yang Li[2], Tianyue Wang[1,2,5], Yu Kang[1,4], Tong Zhu[2], Chang-yu Hsieh[1,4,*], Tingjun Hou[1,4,*]

[1]College of Pharmaceutical Sciences, Zhejiang University, Hangzhou 310058, Zhejiang, China

[2]Shanghai Innovation Institute, Shanghai 200030, China

[3]CarbonSilicon AI Technology Co., Ltd, Hangzhou 310018, Zhejiang, China

[4]Zhejiang Provincial Key Laboratory for Intelligent Drug Discovery and Development, Jinhua 321016, Zhejiang, China

[5]Shanghai Academy of AI for Science, Shanghai 200030, China

E-mail: kimhsieh@zju.edu.cn; tingjunhou@zju.edu.cn

Supplementary Figures

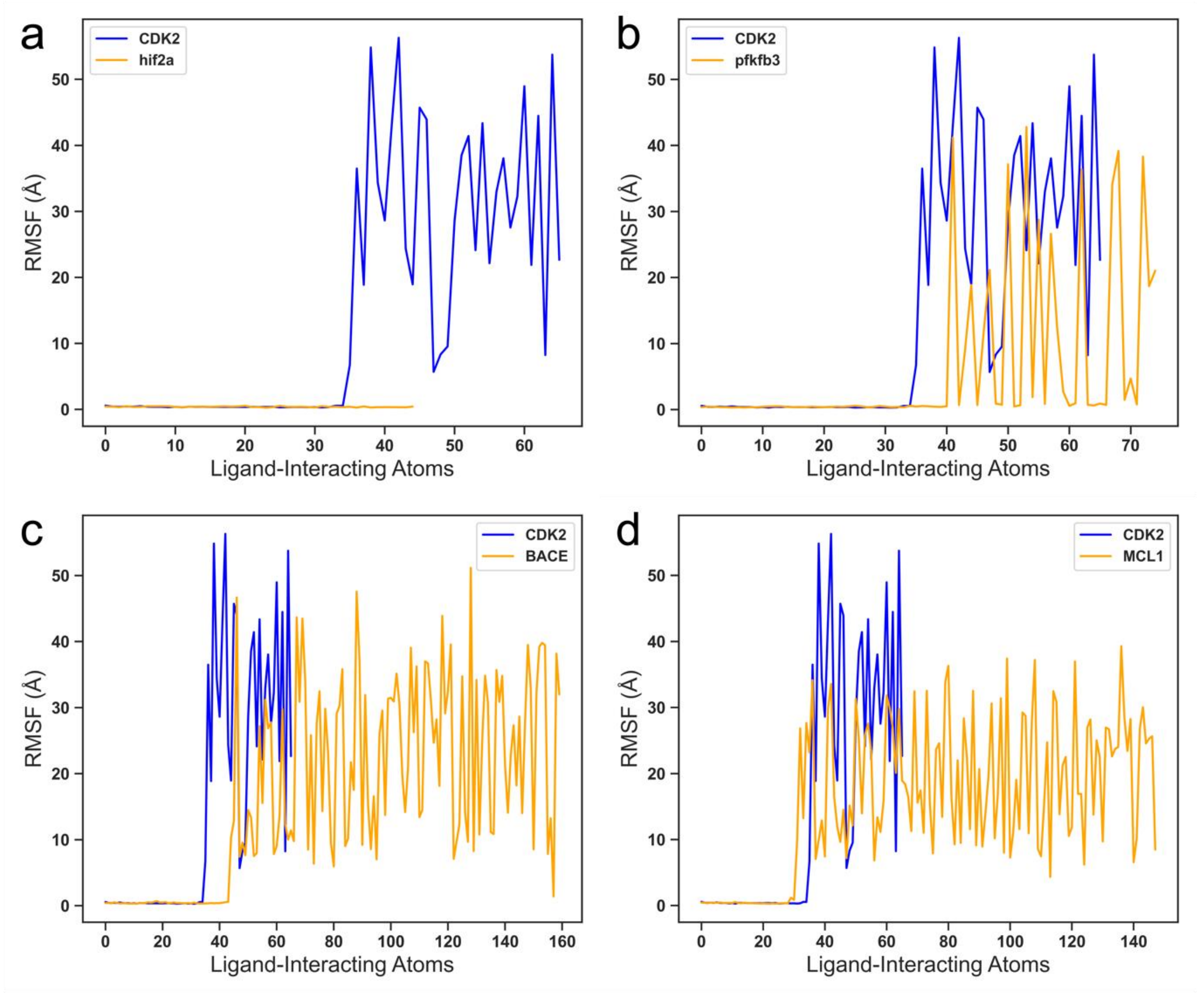

Figure S1. The RMSF of the ligand-interacting atoms within the binding pockets in CDK2 (shown as blue lines) and (a) hif2a, (b) pfkfb3, (c) BACE, and (d) MCL1 (shown as orange lines). The atoms located within 6Å of the ligands are regarded as ligand-interacting.

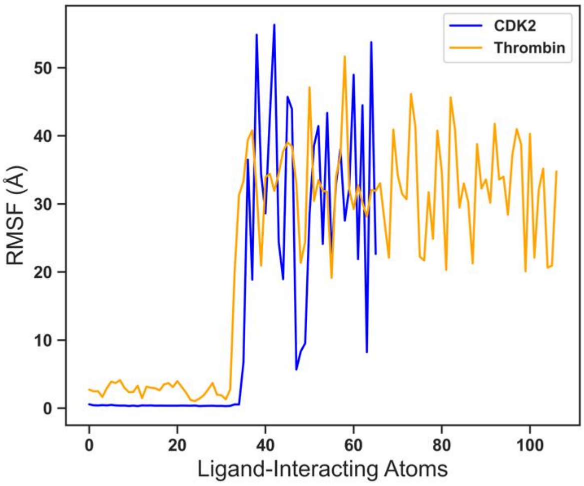

Figure S2. The RMSF of the ligand-interacting atoms within the binding pockets in CDK2 (shown as blue lines) and Thrombin (shown as orange lines). The atoms located within 6Å of the ligands are regarded as ligand-interacting.

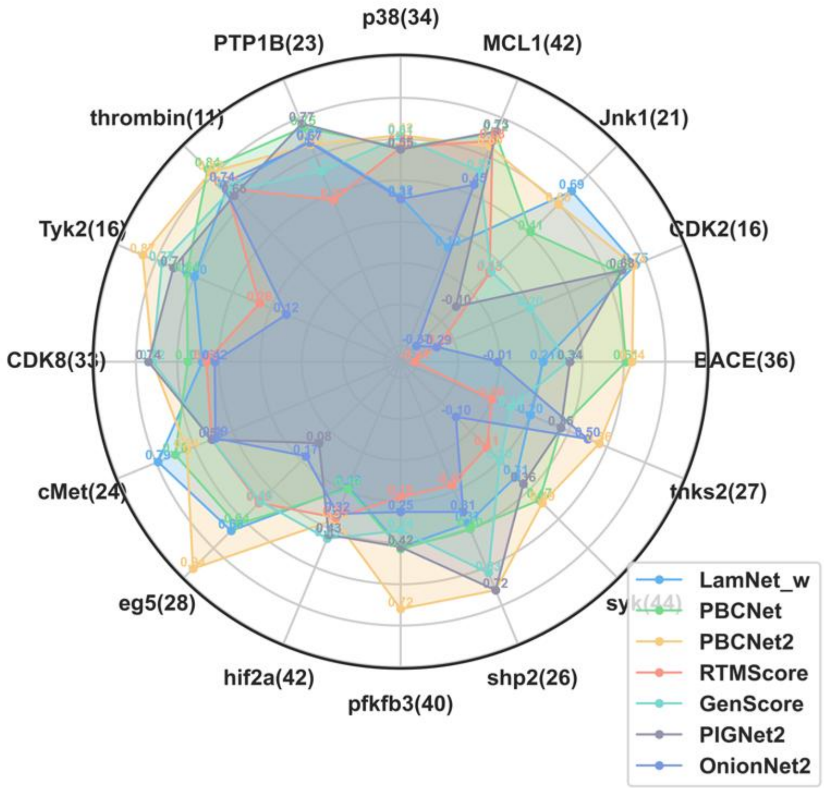

Figure S3. The Pearson r performance on FEP1 and FEP2 benchmarks of LamNet, PBCNet, PBCNet2, RTMScore, GenScore, PIGNet2, and OnionNet2. The results for other models are sourced from the PBCNet2 paper, in which the preprocessing of the initial structures is somehow inconsistent with ours[1].

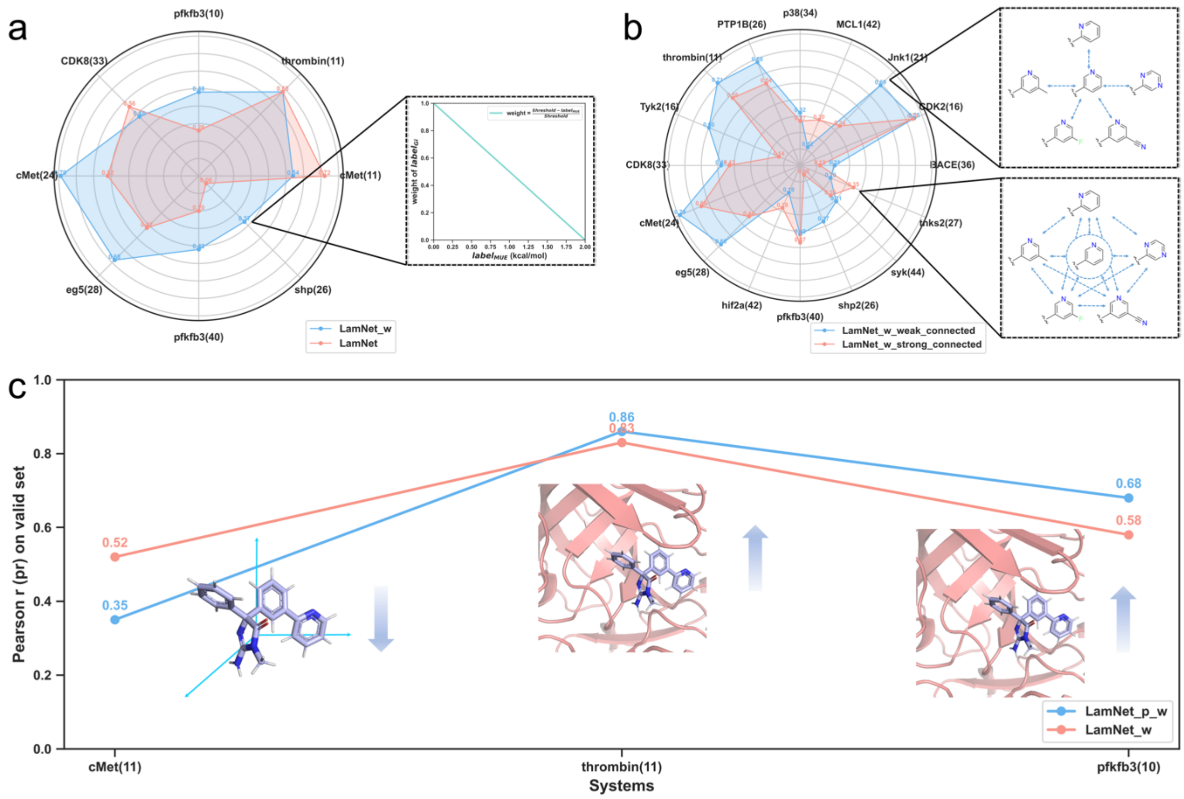

Figure S4. Impact of loss weighting, mapping strategies, and input preprocessing on the Pearson r performance of LamNet. **(a)** The Pearson r performance across 8 systems with and without the loss weighting strategy. **(b)** The Pearson r performance on 16 systems comparing RBFE calculations using weakly connected versus strongly connected mappings. **(c)** The line plot of Pearson r performance across 3 systems with and without input structure preprocessing. The ligand structure underwent preprocessing for maximum substructure alignment on the cMet (11) system, while the complex structures were minimized and pre-equilibrated on thrombin (11) and pfkfb3 (10).

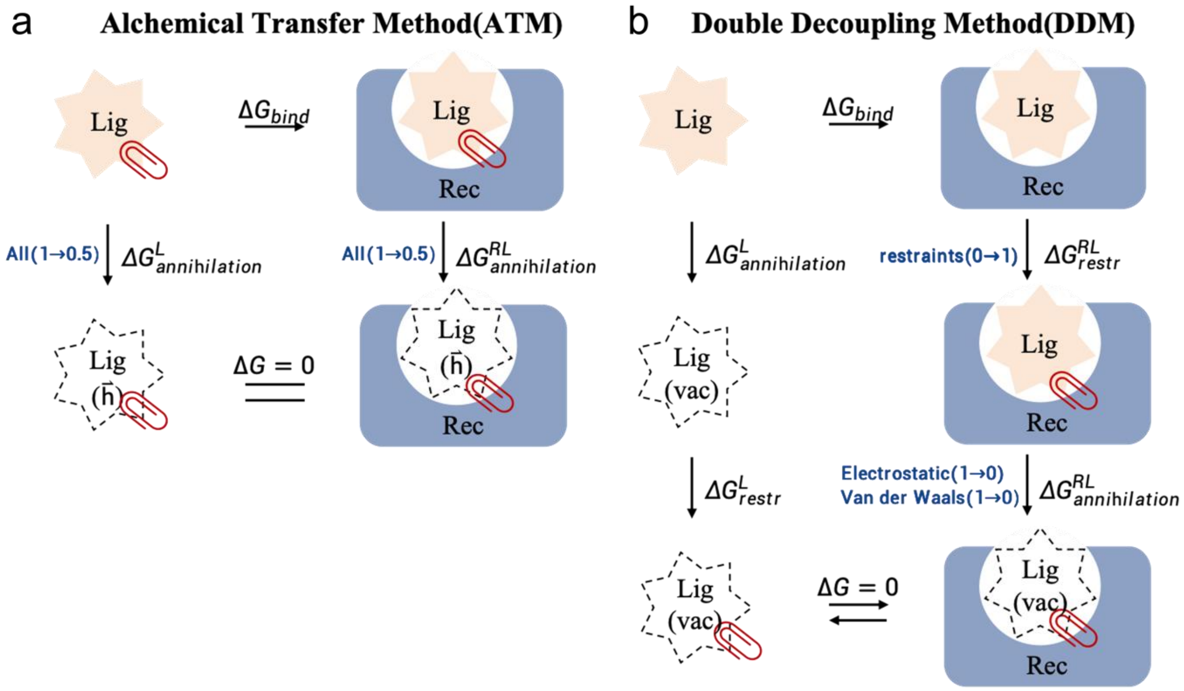

Figure S5. Schematic illustration of the computational principles of ATM (**a**) and conventional AFEMs represented by the double decoupling method (DDM) (**b**).

Supplementary Tables

Table S1: Overview of the RBFE dataset.

Metrics	Systems											
	CDK2	JNK1	TYK2	hif2a	p38	pfkfb3	syk	tnks2	BACE	MCL1	Thrombin	PTP1B
**Crystal structure**[a]	1H1Q	2GMX	4GIH	5TBM	3FLY	6HVI	4PV0	4UI5	4DJW	4HW3	2ZFF	2QBS
**No. of pairs**[b]	47*	57*	48*	28	105*	47	37	45	57	71	16	49
**Method**[c]	ATM-nnp + ATM-mm	ATM-nnp + ATM-mm	ATM-nnp + ATM-mm	ATM-nnp	ATM-nnp + ATM-mm	ATM-nnp	ATM-nnp	ATM-nnp	ATM-mm	ATM-mm	ATM-mm	ATM-mm
**No. of successful calc.**[d]	45	53	47	25	101	47	36	34	57	71	16	48
**No. of pairs with large err./st.dev.**[e]	22	13	18	12	61	27	15	14	24	34	7	26
**No. of failed calc.**[f]	2	4	1	3	4	0	1	11	0	0	0	1
**Ligand pairs split (Train/Valid)**[g]	19:4	33:7	24:5	11:2	33:7	17:3	17:4	16:4	27:6	30:7	7:2	18:4
**Train data size**[h]	418	726	528	242	726	374	374	352	594	660	154	396

[a] The crystal structure used as the reference of binding mode for each protein-ligand dataset.

[b] The total number of ligand pairings computed for each protein-ligand dataset. * The sum of the total of ligand pairs calculated in ATM_MM and ATM_MM/NNP.

[c] The computational methodologies related to this target, including ATM-mm and ATM-NNP.

[d] The number of successful ligand pairs in performing the ATM computation.

[e] The number of ligand pairs exhibiting a MUE greater than 2.0 kcal/mol or an excessively large standard deviation at the time of the ATM calculation.

[f] The number of ligand pairs that failed as a result of system collapse during ATM computations.

[g] The ratio of ligand pairs distributed to training and validation sets at the ligand pair level for each target.

[h] The number of training data points included in each target.

Table S2: Performance comparison of LamNet and its variants with LaDyBUGS and TI across 3 protein-ligand systems.

Systems	Ligand pairs	Metrics	Methods							
			LamNet	LamNet_p	LamNet_w	LamNet_p_w	LaDyBUGS 5ns	LaDyBUGS 15ns	TI 5ns/λ	TI 15ns/λ
**cMet**	11	MUE	**0.66 ± 0.16**	0.80 ± 0.15	0.76 ± 0.16	0.79 ± 0.20	0.97 ± 0.15	0.92 ± 0.13	1.01 ± 0.14	0.88 ± 0.13
		RMSE	**0.84 ± 0.19**	0.94 ± 0.17	0.92 ± 0.17	1.04 ± 0.21	1.09 ± 0.14	1.02 ± 0.12	1.12 ± 0.13	0.98 ± 0.11
		pr	**0.72 ± 0.16**	0.52 ± 0.22	0.54 ± 0.14	0.35 ± 0.22	0.44 ± 0.17	0.51 ± 0.16	0.48 ± 0.16	0.57 ± 0.14
		τ	**0.70 ± 0.16**	0.29 ± 0.25	0.48 ± 0.16	0.22 ± 0.24	0.31 ± 0.16	0.40 ± 0.17	0.26 ± 0.20	0.29 ± 0.18
**thrombin**	10	MUE	0.34 ± 0.09	0.29 ± 0.04	0.45 ± 0.10	**0.23 ± 0.04**	0.89 ± 0.24	0.80 ± 0.21	1.06 ± 0.20	1.07 ± 0.23
		RMSE	0.45 ± 0.10	0.31 ± 0.03	0.56 ± 0.12	**0.26 ± 0.03**	1.19 ± 0.26	1.05 ± 0.24	1.26 ± 0.21	1.31 ± 0.23
		pr	0.68 ± 0.17	0.83 ± 0.08	0.68 ± 0.18	0.86 ± 0.08	0.94 ± 0.03	**0.96 ± 0.02**	0.93 ± 0.04	0.94 ± 0.04
		τ	0.49 ± 0.20	0.56 ± 0.15	0.49 ± 0.22	0.71 ± 0.19	0.75 ± 0.13	**0.82 ± 0.10**	0.82 ± 0.12	0.82 ± 0.12
**pfkfb3**	11	MUE	1.09 ± 0.18	1.01 ± 0.12	0.90 ± 0.21	**0.86 ± 0.17**	1.08 ± 0.11	1.02 ± 0.10	1.42 ± 0.17	1.19 ± 0.14
		RMSE	1.21 ± 0.15	1.07 ± 0.13	1.11 ± 0.24	**1.02 ± 0.22**	1.15 ± 0.10	1.07 ± 0.09	1.52 ± 0.14	1.27 ± 0.11
		pr	0.26 ± 0.34	0.58 ± 0.24	0.48 ± 0.30	0.68 ± 0.12	0.80 ± 0.15	**0.81 ± 0.14**	0.78 ± 0.18	0.80 ± 0.15
		τ	0.33 ± 0.31	0.56 ± 0.25	0.33 ± 0.31	**0.73 ± 0.16**	0.69 ± 0.22	**0.72 ± 0.21**	0.64 ± 0.27	0.63 ± 0.24

The table compares the RBFE prediction performance of LamNet and its variants (LamNet_p, LamNet_w, LamNet_p_w) against LaDyBUGS and TI on three systems: cMet (11), thrombin (10), and pfkfb3 (11). Evaluation metrics include MUE, RMSE, Pearson r (pr), and Kendall's tau (τ). Bold values highlight the best-performing method in each category. Simulation durations for LaDyBUGS and TI are 5 ns and 15 ns (or 5 ns/λ and 15 ns/λ), respectively. The estimated standard deviations of ΔG were obtained by Bootstrapping.

Table S3: Performance comparison of LamNet with FEP/REST and CS-FEP-CAR across FEP1 systems.

Methods	Metrics		Systems								All
			BACE	CDK2	Jnk1	MCL1	p38	PTP1B	thrombin	Tyk2	
No. of ligands			36	16	21	42	34	23	11	16	199
**LamNet**	dG	MUE	0.87 ± 0.12	0.76 ± 0.15	0.92 ± 0.18	1.14 ± 0.14	0.95 ± 0.19	0.87 ± 0.15	0.42 ± 0.10	0.88 ± 0.13	0.91 ± 0.06
		RMSE	1.10 ± 0.16	0.95 ± 0.16	1.26 ± 0.26	1.49 ± 0.17	1.43 ± 0.34	1.15 ± 0.20	0.53 ± 0.11	1.01 ± 0.14	1.24 ± 0.09
		pr	0.21 ± 0.19	0.75 ± 0.08	0.69 ± 0.14	0.12 ± 0.16	0.32 ± 0.14	0.68 ± 0.11	0.71 ± 0.21	0.60 ± 0.20	0.61 ± 0.05
		τ	0.13 ± 0.13	0.58 ± 0.12	0.66 ± 0.12	0.10 ± 0.12	0.33 ± 0.12	0.52 ± 0.11	0.45 ± 0.22	0.38 ± 0.20	0.47 ± 0.04
**CS-FEP-CAR**	dG	MUE	/	0.55 ± 0.16	0.82 ± 0.12	/	/	/	0.49 ± 0.11	0.72 ± 0.15	/
		RMSE	/	0.70 ± 0.21	0.95 ± 0.13	/	/	/	0.59 ± 0.11	0.89 ± 0.15	/
		pr	/	0.78 ± 0.13	0.79 ± 0.10	/	/	/	0.75 ± 0.17	0.50 ± 0.19	/
**FEP/REST 5ns/λ**	dG	MUE	0.67 ± 0.09	0.88 ± 0.14	1.06 ± 0.09	0.84 ± 0.09	0.86 ± 0.08	0.61 ± 0.11	0.42 ± 0.10	0.45 ± 0.09	0.76 ± 0.04
		RMSE	0.85 ± 0.09	1.04 ± 0.13	1.14 ± 0.09	1.04 ± 0.11	0.97 ± 0.09	0.80 ± 0.14	0.54 ± 0.12	0.57 ± 0.11	0.93 ± 0.04
		pr	0.78 ± 0.07	0.48 ± 0.19	0.85 ± 0.07	0.77 ± 0.05	0.65 ± 0.08	0.80 ± 0.08	0.71 ± 0.21	0.89 ± 0.06	0.81 ± 0.02
		τ	0.57 ± 0.09	0.28 ± 0.18	0.76 ± 0.09	0.60 ± 0.07	0.46 ± 0.08	0.69 ± 0.11	0.45 ± 0.23	0.70 ± 0.11	0.62 ± 0.03

This table compares the RBFE prediction accuracy of LamNet, CS-FEP-CAR, and FEP/REST 5ns/λ on 8 protein-ligand systems (BACE, CDK2, Jnk1, MCL1, p38, PTP1B, thrombin, and Tyk2). Evaluation metrics include MUE, RMSE, Pearson r (pr), and Kendall's tau (τ). Slashes ("/") denote missing or inapplicable data. The estimated standard deviations of ΔG were obtained by Bootstrapping.

Table S4: Performance comparison of LamNet with FEP+ across FEP2 systems.

Methods	Metrics		Systems								All
			CDK8	cMet	eg5	hif2a	pfkfb3	shp2	syk	tnks2	
No. of ligands			33	24	28	42	40	26	44	27	264
**LamNet**	dG	MUE	0.86 ± 0.15	0.86 ± 0.17	1.50 ± 0.14	1.02 ± 0.13	1.09 ± 0.15	0.97 ± 0.11	0.66 ± 0.07	1.27 ± 0.17	1.01 ± 0.05
		RMSE	1.23 ± 0.27	1.17 ± 0.23	1.68 ± 0.13	1.34 ± 0.16	1.42 ± 0.22	1.13 ± 0.10	0.81 ± 0.08	1.55 ± 0.17	1.30 ± 0.07
		pr	0.48 ± 0.15	0.79 ± 0.09	0.68 ± 0.09	0.18 ± 0.12	0.42 ± 0.11	0.37 ± 0.13	0.31 ± 0.11	0.20 ± 0.21	0.63 ± 0.03
		τ	0.43 ± 0.10	0.58 ± 0.13	0.55 ± 0.12	0.14 ± 0.10	0.28 ± 0.11	0.22 ± 0.12	0.13 ± 0.10	0.06 ± 0.15	0.48 ± 0.03
**FEP+ 5ns/λ**	dG	MUE	1.20 ± 0.15	0.82 ± 0.11	0.62 ± 0.11	0.84 ± 0.11	1.06 ± 0.09	0.74 ± 0.12	0.85 ± 0.11	1.23 ± 0.18	0.93 ± 0.04
		RMSE	1.45 ± 0.19	0.99 ± 0.10	0.86 ± 0.14	1.12 ± 0.19	1.22 ± 0.10	0.96 ± 0.14	1.12 ± 0.13	1.53 ± 0.17	1.19 ± 0.06
		pr	0.62 ± 0.12	0.90 ± 0.03	0.70 ± 0.08	0.61 ± 0.15	0.79 ± 0.05	0.71 ± 0.08	0.50 ± 0.15	0.40 ± 0.17	0.76 ± 0.03
		τ	0.57 ± 0.09	0.73 ± 0.07	0.54 ± 0.09	0.45 ± 0.10	0.59 ± 0.06	0.61 ± 0.09	0.29 ± 0.10	0.29 ± 0.13	0.57 ± 0.03

This table compares the RBFE prediction accuracy of LamNet and FEP+ 5ns/λ on 8 protein-ligand systems (CDK8, cMet, eg5, hif2a, pfkfb3, shp2, syk, tnks2). Evaluation metrics include MUE, RMSE, Pearson r (pr), and Kendall's tau (τ). The estimated standard deviations of ΔG were obtained by Bootstrapping.

Table S5: Performance comparison of LamNet with various data-driven methods across FEP1 systems.

Methods	Metrics		Systems								Average pr
			BACE	CDK2	Jnk1	MCL1	p38	PTP1B	thrombin	Tyk2	
**LamNet**	dG	MUE	0.87 ± 0.12	0.76 ± 0.15	0.92 ± 0.18	1.14 ± 0.14	0.95 ± 0.19	0.87 ± 0.15	0.42 ± 0.10	0.88 ± 0.13	0.51
		RMSE	1.10 ± 0.16	0.95 ± 0.16	1.26 ± 0.26	1.49 ± 0.17	1.43 ± 0.34	1.15 ± 0.20	0.53 ± 0.11	1.01 ± 0.14	
		pr	0.21 ± 0.19	0.75 ± 0.08	0.69 ± 0.14	0.12 ± 0.16	0.32 ± 0.14	0.68 ± 0.11	0.71 ± 0.21	0.60 ± 0.20	
		τ	0.13 ± 0.13	0.58 ± 0.12	0.66 ± 0.12	0.10 ± 0.12	0.33 ± 0.12	0.52 ± 0.11	0.45 ± 0.22	0.38 ± 0.20	
**PBCNet**	dG	pr	0.61	0.66	0.41	0.72	0.56	0.75	0.84	0.64	0.65
**PBCNET2**	dG	pr	0.64	0.74	0.60	0.65	0.62	0.66	0.82	0.87	0.70
**RTMScore**	dG	pr	-0.41	-0.28	0.13	0.68	0.56	0.37	0.71	0.26	0.25
**GenScore （GatedGCN_ft_1.0）**	dG	pr	0.33	0.2	0.14	0.52	0.61	0.52	0.69	0.77	0.47
**PIGNet2**	dG	pr	0.34	0.68	-0.10	0.73	0.55	0.77	0.66	0.71	0.54
**OnionNet2**	dG	pr	-0.01	-0.29	-0.37	0.45	0.31	0.67	0.74	0.12	0.20

This table compares the RBFE prediction accuracy of LamNet, PBCNet, PBCNet2, RTMScore, GenScore (GatedGCN_ft_1.0), PIGNet2, and OnionNet2 on 8 protein-ligand systems (BACE, CDK2, Jnk1, MCL1, p38, PTP1B, thrombin, and Tyk2). Evaluation metrics include Pearson r (pr). The estimated standard deviations of ΔG were obtained by Bootstrapping.

Table S6: Performance comparison of LamNet with various data-driven methods across FEP2 systems.

**Methods**	**Metrics**		**Systems**								**Average pr**
			CDK8	cMet	eg5	hif2a	pfkfb3	shp2	syk	tnks2	
**LamNet**	dG	MUE	0.86 ± 0.15	0.86 ± 0.17	1.50 ± 0.14	1.02 ± 0.13	1.09 ± 0.15	0.97 ± 0.11	0.66 ± 0.07	1.27 ± 0.17	0.47
		RMSE	1.23 ± 0.27	1.17 ± 0.23	1.68 ± 0.13	1.34 ± 0.16	1.42 ± 0.22	1.13 ± 0.10	0.81 ± 0.08	1.55 ± 0.17	
		pr	0.48 ± 0.15	0.79 ± 0.09	0.68 ± 0.09	0.18 ± 0.12	0.42 ± 0.11	0.37 ± 0.13	0.31 ± 0.11	0.20 ± 0.21	
		τ	0.43 ± 0.10	0.58 ± 0.13	0.55 ± 0.12	0.14 ± 0.10	0.28 ± 0.11	0.22 ± 0.12	0.13 ± 0.10	0.06 ± 0.15	
**PBCNet**	dG	pr	0.55	0.70	0.64	0.19	0.43	0.40	0.47	0.36	0.47
**PBCNET2**	dG	pr	0.72	0.64	0.94	0.35	0.72	0.72	0.49	0.56	0.63
**RTMScore**	dG	pr	0.46	0.51	0.49	0.34	0.18	0.17	0.11	0.00	0.28
**GenScore （GatedGCN_ft_1.0）**	dG	pr	0.72	0.51	0.48	0.45	0.34	0.63	0.20	0.10	0.43
**PIGNet2**	dG	pr	0.74	0.51	0.08	0.43	0.42	0.72	0.36	0.36	0.45
**OnionNet2**	dG	pr	0.42	0.49	0.17	0.32	0.25	0.31	-0.1	0.5	0.30

This table compares the RBFE prediction accuracy of LamNet, PBCNet, PBCNet2, RTMScore, GenScore (GatedGCN_ft_1.0), PIGNet2, and OnionNet2 on 8 protein-ligand systems (CDK8, cMet, eg5, hif2a, pfkfb3, shp2, syk, tnks2). Evaluation metrics include Pearson r (pr). The estimated standard deviations of ΔG were obtained by Bootstrapping.

Table S7: Overview of the ABFE dataset.

Metrics	System[a]														
	CBn-type					octa-acid		Gibb deep carvity cavitand							Pillararene
	4CB7	5CBClip	6CB8	7trimertrip	8CB8	9HDM-bCD	9bCD	5TEMOA	5OA	6TEMOA	6OA	7exoOA	7OA	8TEMOA	9WP6
**No. of guests**[b]	15	10	14	16	7	5	5	6	6	8	8	8	8	5	13
**No. of successful calc.**[c]	14	5	12	11	6	5	5	6	6	8	8	6	7	5	10
**no. of pairs with large err./st.dev.**[d]	11	2	7	10	4	4	0	5	3	3	1	6	5	3	10
**No. of failed calc.**[e]	1	5	2	5	1	0	0	0	0	0	0	2	1	0	3
**No. of calc. in dataset**[f]	3	1	5	1	2	1	5	1	3	5	7	0	2	2	0
**data size**[g]	264					132		440							0
**Ligand split (Train/Valid)**[h]	31:7														

[a] The type of host pocket, classified into 4 types.

[b] The total number of guests computed for each host-guest dataset.

[c] The number of successful guests in performing the ATM computation.

[d] The number of guests exhibiting a MUE greater than 3.0 kcal/mol or an excessively large standard deviation at the time of the ATM calculation.

[e] The number of guests that failed as a result of system collapse during ATM computations.

[f] The number of guests that are involved in our ABFE dataset.

[g] The number of training data points included in each type of host pocket.

[h] The ratio of guests distributed to training and validation sets at the guest level for the whole dataset.

Table S8: Hyperparameter settings of LamNet under different training modes and task type.

Training Mode	Task Type	Description	Learning Rate (lr)	Weight Decay (wd)	Batch Size	Weighting Strategy
multi	RBFE	Multi-target joint traning	1.00E-02	1.00E-03	32	AUE-weighted (use_aue_weight=True)
multi	ABFE	Multi-host joint traning	1.00E-02	1.00E-03	32	No weighting (use_aue_weight=False)
single	RBFE	Single-target training (e.g.,CDK2)	5.00E-04	5.00E-03	16	AUE-weighted (use_aue_weight=True)
fewshot	RBFE	Few-shot traning (e.g.,PTP1B)	1.00E-02	1.00E-03	32	AUE-weighted (use_aue_weight=True)

This table summarizes the descriptions, learning rates (lr), weight decay values (wd), batch sizes, and weighting strategies used in LamNet training under various modes and task types, including multi-target-RBFE, multi-host-ABFE, single-target-RBFE, and RBFE few-shot learning. The use_aue_weight=True in the weighting strategy indicates that the MUE of ΔG was used to weight the loss during training.

Source data and specific details of Figures 2-6 and Table 1 (see SI-data.xlsx)

Statistical source data.

Supplementary Context

Datasets

Utilizing the ATM, an innovative AFEM, we calculated and collected our RBFE and ABFE datasets.

RBFE datasets. Two sets of calculations have been made for the RBFE dataset, denoted as ATM_MM and ATM_MM/NNP. The reason for preparing ATM_MM was primarily practical, reflecting the limits of our accessible time and cost. By contrast, ATM_MM/NNP was prepared because the hybrid ML/MM force field provides a more precise description of ligand interactions and thus achieves higher accuracy. ATM_MM contains 8 protein targets: BACE, CDK2, JNK1, MCL1, p38, thrombin, TYK2, and PTP1B[2]. The protein force field applied is Amber ff14SB[3], the explicit water model is TIP3P, and the ligand charge assignment and force field employ AM1-BCC and GAFF2[4], respectively. ATM_MM/NNP comprises 8 protein targets: CDK2, JNK1, TYK2, hif2a, p38, pfkfb3, syk, and tnks2[5]. The protein force field, the explicit water force field, and the ligand charge assignment are utilized in the same way as described above. However, the ligand inner was modeled with the ANI-2x NNP[6], whereas the ligand-environment interaction was modeled with GAFF2.

The perturbation network and simulation procedure implemented in both calculation sets align with those reported by Zariquiey et al.[2,5] All inputs for the simulation system can be directly accessed at https://github.com/compsciencelab/ATM_benchmark. All simulations were performed with the ASyncRE software and the ATM MetaForce plugin within the OpenMM engine, employing Hamiltonian replica exchange to improve convergence[2]. To reduce data collecting costs, each pair of ligands was simulated using 22 evenly spaced λ-windows, each sampling 1 ns. ATM_MM and ATM_MM/NNP time-steps were 2 fs and 4 fs, respectively. Bond lengths involving hydrogens were constrained using the CCMA algorithm in OpenMM[7], and hydrogen mass repartitioning was applied in the ATM_MM/NNP

simulations to enable a larger time step. Furthermore, all simulations utilized the ATMMTSLangevinIntegrator, which supports multiple time-step integration of the potential while maintains accuracy and stability at a larger outer timestep[8]. Simulations were executed with a single NVIDIA A100 GPU for ATM_MM and a single NVIDIA H100 GPU for ATM_MM/NNP. The ATM_MM/NNP simulation required approximately 3 days every ligand pair, whereas the ATM_MM simulation necessitated just about 1.5 days per ligand pair. A total of 607 ligand pairs were calculated for the RBFE dataset. Specific details of the computational information are available in the **Refs 5 and 2**. The estimated window-level energies (Gi), ΔΔG, and their corresponding standard deviations were derived with UWHAM. The window-level free energies and mean unsigned errors (MUE) of ΔΔG were collected for each pair of ligands.

To enhance the quality of the training data, we excluded ligand pairs with an MUE of ΔΔG over 2.0 kcal/mol and those who suffered computational failures. A total of 307 ligand pairs have been collected, resulting in 6754 points of data. To precisely quantify the model's generalization performance in the experiments, we carefully and rigorously divided the training and validation sets at the ligand pair level, ensuring that the ligand pairs in the validation set were absent from the training set. Specifically, when targets were present in both the ATM_MM and ATM_MM_NNP datasets (e.g., CDK2, JNK1, TYK2, p38), additional care was taken during train/validation splitting to prevent data leakage, satisfying the requirement mentioned above while maintaining balanced and representative validation sets across both datasets. This ensures a rigorous division of the dataset for future learning and assessment. The total amount of available training data post-delineation was 5478. Comprehensive details regarding the ligand pair calculated for each target in the RBFE dataset are provided in **Table S1**.

To obtain the model inputs for each point of data, ligand-pocket complex generation processing was conducted on the simulated systems in the ATM_MM and ATM_MM/NNP datasets. For ATM_MM, the protein and ligand structures used to build the simulation inputs have been included separately and can be accessed at https://github.com/compsciencelab/ATM_benchmark. The process involved (1) ligand standardization: employing OpenBabel[9] to unify and standardize the ligand format to either

sdf or pdb; (2) protein standardization: utilizing OpenBabel to standardize the protein format to pdb, and applying Biopython[10] to convert non-standardized protein residues to their standardized forms; (3) protein pocket extraction: utilizing PyMOL[11] to extract residues within 5.0 Å of the ligand, excluding water and hydrogen, to define binding pockets; and (4) ligand-pocket complex generation: the standardized ligand and extracted pockets were merged into an RDKit Mol object[12] and subsequently saved as a .rdkit file after validation. However, for ATM_MM/NNP, only simulated systems are provided at https://github.com/compsciencelab/ATM_benchmark. Therefore, the simulated system was parsed with Biopython to identify and separate protein and ligand structures before the aforementioned pre-processing. It should be noted that, considering that the reference ligand in ATM's RBFE simulation system is typically situated within the protein pocket while the perturbing ligand typically lies at the outer boundary of the periodic box via a position vector, the alignment of the two ligands within the protein pocket was accomplished using RDKit's MCS algorithm and least-squares for rigid-body rotation alignment. Further processing steps are the same as those used for ATM_MM.

ABFE datasets. The ABFE dataset was obtained by performing ABFE calculations on 15 different host-guest systems from SAMPL4 to SAMPL9 challenges[13–18]. These hosts covered four classes based on the type of pocket: Pillararene, Gibb deep carvity cavitand, octa-acid, and CBn-type. The structural files for both the host and guest are available for download from the GitHub repository corresponding to the challenges. After downloading, the guest is firstly docked into the host using AutoDock Vina[19]. Considering that the majority of the pockets are situated near the center of mass of the host, we assign the initial point of the docked grid as the center of mass of the host, with a default size of 10 for docking. Subsequently, we utilized the LEaP program in AmberTools[20] to set up the simulation system. Then HTMD[21] was utilized to identify heavy atoms that engaged in the restraint potential, and the ligand displacement vector was defaulted to 22 Å. The host and guest force fields utilized were GAFF2, with the charge assignment method being AM1-BCC for both, while the explicit water force field employed was TIP3P[4]. It should be noted that we initially excluded the host-guest pairs that could not be docked successfully with AutoDock Vina, along with those that could not be parameterized by GAFF2.

In order to ensure the availability of training data while reducing computing expenses, each ABFE computation utilized 22 uniformly distributed λ-windows, each sampled at 10 ns, with a time step of 1 fs. The simulations were executed on a single NVIDIA H100 GPU, with each pair of host-guest taking approximately 2 days of wall time. The ABFE calculation was carried out to 134 host-guest pairs in total. Specific details of the calculations are fully located in **Refs 8 and 27**. The estimated window-level energies (Gi), ΔG, and their corresponding standard deviations were calculated with UWHAM. For each host-guest pair, we collected the window-level free energies and the MUE of ΔΔG.

Similarly, to ensure the optimal quality of the training data, we excluded host-guest pairs with an MUE of ΔG over 3.0 kcal/mol and those that encountered computational failures. A total of 38 host-guest pairs were obtained, resulting in 836 points of data. To verify that the model's generalization performance has been precisely captured in the experiments, we carefully and rigorously divided the training and validation sets at the guest level, guaranteeing that the guests in the validation set are not present in the training set. The total amount of available training data post-division is 682. Comprehensive details about the computation of the ABFE dataset are included in **Table S7**.

Beyond dataset-level processing, model input processing, which involves preparing λ values together with the corresponding three-dimensional (3D) structures for each entry of data, is basically identical to that mentioned in the previous RBFE section. The key differences are (1) the initial configurations of the host and guest are the structure obtained after AutoDock Vina docking; (2) the identification of host-guest pockets within a 5.0 Å range is performed on an atom-by-atom basis rather than by residues; and (3) the generation of RDKit Mol objects for the guest has been incorporated alongside the RDKit Mol object generation for the guest-pocket complexes.

Model Architecture

Input. LamNet uses 3D structures (ligand-pocket complexes or single ligands) and λ parameters as inputs, with the protein pocket defined as all residues within 5 Å of the ligand. For RBFE (see **Figure 1b1**), two ligand-pocket complexes and λ parameters are used as model input. For ABFE (see Figure 1b2), one ligand-pocket complex, one ligand and λ parameters are used as

model input. LamNet depicts a ligand-pocket complex as a 3D heterogeneous interaction graph:

$$\mathcal{G} = (\mathcal{V}, \mathcal{E}, \mathcal{R}) = \left(\mathcal{V}_\ell \cup \mathcal{V}_p, \mathcal{E}_\ell \cup \mathcal{E}_p \cup \mathcal{E}_{\ell p}, \mathcal{R}_\ell \cup \mathcal{R}_p\right) \#(1)$$

where $\mathcal{V}_\ell$ and $\mathcal{V}_p$ correspond to the nodes (atoms) of the ligand and pocket, respectively, $\mathcal{E}_\ell$ and $\mathcal{E}_p$ correspond to the covalent interaction edges in the ligand and pocket, respectively, $\mathcal{E}_{\ell p}$ corresponds to the non-covalent interaction edges between the ligand-pocket complexes, and $\mathcal{R}_\ell, \mathcal{R}_p \subset R^3$ corresponds to the 3D coordinates of the ligand and pocket, respectively. Each node $v_i \in \mathcal{V}$ is associated with an initial node feature vector $x_i \in R^n$ and a spatial coordinate $r_i \in R^3$. A covalent edge $e_{ij} \in \mathcal{E}_\ell \cup \mathcal{E}_p$ exists if there is a chemical bond between atoms v_i and v_j. A non-covalent edge $e_{ij} \in \mathcal{E}_\ell \cup \mathcal{E}_p$ exists if the distance between ligand atoms $v_i \in \mathcal{V}_\ell$ and pocket atoms $v_k \in \mathcal{V}_p$ is less than 5 Å ($d_{ik} = |r_i - r_k|_2 < 5$). Similarly, given a ligand, LamNet encodes it as a 3D non-heterogeneous interaction graph:

$$\mathcal{G} = (\mathcal{V}, \mathcal{E}, \mathcal{R}) = (\mathcal{V}_\ell, \mathcal{E}_\ell, \mathcal{R}_\ell) \#(2)$$

where $\mathcal{V}_\ell$ corresponds to the nodes (atoms) of the ligand, $\mathcal{E}_\ell$ corresponds to the covalent interaction edges in the ligand and $\mathcal{R}_\ell \subset R^3$ corresponds to the 3D coordinates of the ligand. Each node $v_i \in \mathcal{V}$ is associated with and initial feature $x_i \in R^d$ and a spatial coordinate $r_i \in R^3$。A covalent edge $e_{ij} \in \mathcal{E}_\ell \cup \mathcal{E}_p$ exists if there is a chemical bond between atoms v_i and v_j. Additionally, given the λ parameter, LamNet encodes it as a scalar auxiliary descriptor e, as part of the feature embedding. To be specific, λ is embedded into a higher-dimensional continuous space to enhance the model's ability to capture nonlinear and complex dependencies along the alchemical pathway, without introducing discrete noise.

Representation learning. Given a well-constructed 3D interaction graph $\mathcal{G} = (\mathcal{V}, \mathcal{E}, \mathcal{R})$, LamNet initially obtains chemical and spatial node embeddings via a multilayer message-passing neural network[22]. The objective of this step for the ligand-pocket complex graph is to discern the distinction between covalent and non-covalent interactions. For the sake of clarity, the subsequent representations are exclusively ligand-pocket complex graphs, with

those of ligand graphs that could be derived easily. At layer t of the message passing, the covalent and non-covalent message acquired by node v_i is defined as follows:

$$m_{ji}^{cov} = M^{cov}\left(h_j^{(t)}, h_i^{(t)}, \lambda(|r_j - r_i|^2)\right), \quad \forall e_{ji} \in \mathcal{E}_\ell \cup \mathcal{E}_{\wp} \#(3)$$

$$m_{ki}^{ncov} = M^{ncov}\left(h_k^{(t)}, h_i^{(t)}, \lambda(|r_k - r_i|^2)\right), \quad \forall e_{ki} \in \mathcal{E}_{\ell\wp} \#(4)$$

where M^{cov}和M^{ncov} represents the covalent and noncovalent message functions, respectively:

$$M^{cov}(h_j, h_i, rbf) = h_j \odot rbf \#(5)$$

$$M^{ncov}(h_k, h_i, rbf) = h_k \odot rbf \#(6)$$

where $\odot$ stands for elemental multiplication and $rbf(\cdot)$ stands for the Gaussian radial basis functions (RBF):

$$rbf(d) = \left[\exp\left(-\frac{(d-\mu_k)^2}{\sigma^2}\right)\right]_{k=1}^{K}, \quad \mu_k \in [0,6], \sigma = \frac{2}{3} \#(7)$$

where d denotes the inter distance between two nodes.

The d_{ji} is transformed and activated by MLP to obtain a vector of coordinate-dependent weights:

$$r_{ji}^{cov} = \mathrm{SiLU}\left(W^{cov} \cdot rbf(d_{ji})\right) \#(8)$$

$$r_{ki}^{ncov} = \mathrm{SiLU}\left(W^{ncov} \cdot rbf(d_{ki})\right) \#(9)$$

and thus, the message vectors corresponding to covalent and noncovalent are respectively:

$$m_{ji}^{cov} = h_i \odot r_{ji}^{cov} \#(10)$$

$$m_{ki}^{ncov} = h_i \odot r_{ki}^{ncov} \#(11)$$

The vector after integrating all the neighbor message is:

$$m_i^{cov} = \sum_{v_j \in \mathcal{N}(v_i)^{cov}} m_{ji}^{cov} \#(12)$$

$$m_i^{ncov} = \sum_{v_k \in \mathcal{N}(v_i)^{ncov}} m_{ki}^{ncov} \#(13)$$

The node embedding is updated by the following formula:

$$h_i^{(t+1)} = \mathrm{BN}\left(\mathrm{LeakyReLU}\left(\mathrm{MLP}_{\mathrm{cov}}\left(h_i^{(t)}, m_i^{\mathrm{cov}}\right) + \mathrm{MLP}_{ncov}\left(h_i^{(t)}, m_i^{\mathrm{ncov}}\right)\right)\right)\#(14)$$

where MLP represents Multilayer Perceptron and BN represents Batch Normalization.

In the specific implementation, given the input ligand-pocket complex graph, LamNet initially projects the feature x_i of a node through a linear transformation layer W_{node}:

$$h_i^{(0)} = \mathrm{SiLU}(W_{\mathrm{node}} \cdot x_i)\#(15)$$

Subsequently, it passes into a three-layer Heterogeneous Interaction Layer (HIL) graph convolution layer:

$$h^{(1)} = \mathrm{GConv}_1\left(h^{(0)}\right)\#(16)$$

$$h^{(2)} = \mathrm{GConv}_2\left(h^{(1)}\right)\#(17)$$

$$h^{(3)} = \mathrm{GConv}_3\left(h^{(2)}\right)\#(18)$$

In each layer, the graph convolution module performs the aforementioned message passing and embedding update mechanism (**Equations 3-14**). The node embeddings output from the final layer are globally summed and pooled to obtain the molecular level of the entire complex:

$$g_{mol} = \mathrm{GlobalAddPool}(\{h_i^T\})\#(19)$$

The whole process is applied to the two endpoints in the perturbation separately to obtain a graph-level representation of the paired inputs $g_1, g_2 \in \mathrm{R}^{\mathrm{d}}(d = 256)$. To incorporate the transformation information of the alchemical path, LamNet further introduces an auxiliary scalar feature to represent the parameter $\lambda \in [0,1]$ and embeds it as contextual information in the model representation learning. Specifically, the alchemical parameter λ is first projected as an auxiliary descriptor $e \in \mathrm{R}^{\mathrm{k}}(k = 1)$ and further mapped to the feature space R^{d} via a learnable weight matrix $W \in \mathrm{R}^{\mathrm{k \times d}}$:

$$\tilde{e} = e \cdot W\#(20)$$

Following that, to convey the transformation message represented by λ, LamNet utilizes a symmetric yet directionally sensitive feature modulation approach: it multiplies $\tilde{\mathrm{e}}$ and its complement $(1 - \tilde{\mathrm{e}})$ by the two endpoint embeddings x_1 and x_2, respectively:

$$\widehat{g_1} = g_1 \odot (1 - \tilde{e})\#(21)$$

$$\widehat{g_2} = g_2 \odot \tilde{e} \#(22)$$

where $\odot$ stands for elemental multiplication. The design encapsulates the physical implications of alchemical transformations: as λ increases, the interaction of one complex progressively intensifies while the other diminishes, so facilitating a dynamic weighing of the contribution of the two states. Thereafter, LamNet integrates these two modulated embedding vectors together with their discrepancies into a conclusive representation[23]:

$$h = [\widehat{g_1}, \widehat{g_2}, \widehat{g_1} - \widehat{g_2}], h \in \mathrm{R}^{3*d} \#(23)$$

The sequence of vector splicing guarantees the model's capacity to recognize the directionality of the state inside the transformation pathway, thereby adhering to the fundamental principle of the transformation in RBFE calculations. Finally, the input to the multilayer feed-forward neural network $\mathcal{F}_{FNN}$ is utilized to extract deeper interaction information via the feature fusion network as follows:

$$\mathrm{z_1} = \mathrm{Dropout(ReLU(W_1 \cdot h + b_1))}, \quad \mathrm{W_1} \in \mathrm{R}^{2d \times 3d}, \ \mathrm{z_1} \in \mathrm{R}^{2d} \#(24)$$

$$\mathrm{z_2} = \mathrm{ReLU(W_2 \cdot z_1 + b_2)}, \quad \mathrm{W_2} \in \mathrm{R}^{d \times 2d}, \ \mathrm{z_2} \in \mathrm{R}^{d} \#(25)$$

$$\mathrm{z_3} = \mathrm{ReLU(W_3 \cdot z_2 + b_3)}, \quad \mathrm{W_3} \in \mathrm{R}^{0.5d \times d}, \ \mathrm{z_3} \in \mathrm{R}^{0.5d} \#(26)$$

$$z = W_4 \cdot z_3 + b_4, \quad W_4 \in R^{d \times 0.5d}, \ z \in R^{d} \#(27)$$

Output（Training process）. The final prediction is done by a three-layer MLP($\mathcal{F}_{\mathrm{FC}}$):

$$\hat{y} = \mathcal{F}_{\mathrm{FC}}(z) \in \mathrm{R}^{1} \#(28)$$

where $\hat{y}$ denotes the window-level free energy. The training loss employs a weighted mean squared error (MSE). A dynamic weighting strategy is implemented to enhance the model's learning from high-quality data (in the context of RBFE):

$$\mathcal{L}_{\text{weighted-MSE}} = \frac{1}{N}\sum_{i=1}^{N} w_i(\widehat{y_i} - y_i)^2 \#(29)$$

where $w_i = (2 - u_i)/2$ represents the weight determined by the accuracy of the training data, where u_i denotes the MUE label associated with $\Delta\Delta\mathrm{G}$ for each point of data. The final loss comprises the weighted $\mathcal{L}_{\mathrm{MSE}}$ outlined previously, in addition to a L_2 regularization term to mitigate overfitting:

$$\mathcal{L}_{\text{total}} = \mathcal{L}_{\text{weighted-MSE}} + b\sum_{j} | \theta_j |_2 \#(30)$$

where $b = 0.005$ is the weight decay coefficient and θ_j is the model learnable parameter.

The training process employs a combination of the AdamW optimizer[24], a linear warm-up for the initial 50 epochs, and the ReduceLROnPlateau[25] strategy for dynamic learning rate adjustment. The early-stopping strategy has been used to reduce training cost and avoid overfitting. To be specific, the training termination criterion is 100 epochs without enhancement in performance. The assessment of performance enhancement primarily relies on the accuracy of window-level energy predictions for the intermediate state (λ = 0.5 in ATM) to guarantee the model's effectiveness in the RBFE and ABFE prediction tasks. Furthermore, we implement customized training strategies for various applications and learning modes. In multi-target training, we utilized a comparatively high learning rate and low weight decay to improve generalization capacity. On the other hand, single-target training utilizes a lower learning rate and higher weight decay to augment convergence stability. In addition, a loss weight strategy based on MUE is utilized in the RBFE application to emphasize the learning of more reliable training samples. Specific parameters and their utilization are detailed in **Table S8** and on GitHub (https://github.com/RenlingHu/LamNet).

Analysis of invariance of LamNet. LamNet is explicitly designed to be invariant to translations and rotations. For any translation vector $t \in R^{3}$ and rotation matrix $Q \in R^{3\times 3}$ with $Q^{\top}Q = I$, the transformated coordinates are $r_i' = Qr_i + t$. Since interatomic distance are preserved:

$$\left|\left(r_j + t\right) - \left(r_i + t\right)\right|^2 = \left|r_j - r_i\right|^2 \#(31)$$

$$\left|\mathrm{Q}\boldsymbol{r_j} - \mathrm{Q}\boldsymbol{r_i}\right|^2 = \left|\boldsymbol{r_j} - \boldsymbol{r_i}\right|^2 \#(32)$$

Therefore, the Gaussian RBF (**Eq. 7**) and the subsequent covalent and noncovalent messages (**Eqs. 5–6**) remain unchanged. Consequently, the aggregated messages (**Eqs. 12–13**) and node updates (**Eq. 14**) are also invariant. As the initial projection (**Eq. 15**), graph convolutions (**Eqs. 16–18**), and global pooling (**Eq. 19**) depend only on these invariants, the molecular embeddings are unaffected by translation and rotation. The subsequent λ-modulation (**Eqs. 20–23**) and feature fusion network (**Eqs. 24–27**) act in feature space without explicit coordinate dependence, and thus preserve this property. Therefore, the entire

representation learning phase of LamNet (**Eqs. 5–27**) is invariant to global translation and rotation.

Application

Relative binding affinity prediction. LamNet calculates the RBFE by predicting the window-level energy for the intermediate state of ligand 1 and ligand 2 binding to the protein (λ = 0.5 in ATM):

$$\Delta\Delta G_{calc} = \Delta G_{0.5}^{leg2} - \Delta G_{0.5}^{leg1} \#(33)$$

ΔG (the calculated absolute binding free energy) of one certain ligand was obtained by:

$$\Delta G_{\text{calc}} = \Delta\Delta G_{\text{calc}} - \left(\frac{\sum \Delta\Delta G_{\text{calc}} - \sum \Delta G_{\text{exp}}}{n}\right) \#(34)$$

where ΔG_{calc} is the calculated ABFE, and ΔG_{exp} is the experimental absolute binding affinity. **Equation 32** refers to using all experimental values of the ligands in the whole protein-ligand system as a reference, rather than fixing a specific ligand as a reference, as was done previously[26]. This approach helps to eliminate the propagation of errors that may exist in any one experimental value.

Absolute binding affinity prediction. LamNet calculates ABFE by predicting the window-level energy of the intermediate state of the ligand and ligand-receptor complex (λ = 0.5 in ATM):

$$\Delta G_{\text{calc}} = \Delta G_{0.5}^{leg2} - \Delta G_{0.5}^{leg1} + 0.87 + \Delta G_{\text{site}} \#(35)$$

where ΔG_{site} is the free energy of transfer of a ligand to the solvent from an ideal solution with a concentration of C° and a volume of binding pocket, and 0.87 is the corrective term used by ATM in calculating the ABFE via UWHAM[8,27].

Alchemical parameter optimization (Overlap between alchemical windows). LamNet determines whether the overlap between the windows is sufficient for the simulation to converge by the free energy difference between the two windows:

$$\Delta G_{ij} = G_i - G_j \#(36)$$

Specifically, drawing inspiration from Zeng et al.[28], LamNet calculates the free energy difference ΔG_{ij} between adjacent alchemical states λ_i and λ_j utilizing the learned molecular representations, with ΔG_{ij} defined as $G_i - G_j$. An empirical threshold of 10 kcal/mol has

been set to evaluate if adequate phase space overlap exists, since large energy differences between adjacent states may hinder convergence. The model examines the whole λ range [0, 1] at an interval of 0.001 to pinpoint areas where swift energy fluctuations require a more intense λ setup. In a nutshell, LamNet introduces an adaptive λ strategy for each ligand-protein system by integrating window-level energy prediction with the λ optimization strategy.

Benchmarks

LaDyBUGS benchmark. This study started with three systems: cMet (10), thrombin (11), and pfkfb3 (10), which were studied with LaDyBUGS published by Robo et al.[29], to assess the accuracy and efficiency of LamNet for RBFE prediction, with a particular emphasis on the variations in initial structure preprocessing strategies for the model. The discourse over the weighting approach is also included (see **Supplementary Information**). It should also be mentioned that the ligand MCS alignment conducted for cMet (10) in the discussion was done with our previously developed ligand internal and Cartesian coordinate reconstruction-based alignment algorithm (LIC-align). The system minimization and equilibration conducted for thrombin (11) and pfkfb3 (10) align with the system's setup during ATM calculations.

FEP1 and FEP2 benchmark. This study utilizes the classical large-scale FEP1 and FEP2 datasets, published by Wang et al. and Schindler et al.[26,30], as secondary test sets to further assess the generalization capability and practical applicability of LamNet for RBFE prediction. The discussion primarily focuses on the variations in the perturbation map connections, while some targets are also involved in the weighting strategy (see **Supplementary Information**). The original benchmark structures of the FEP1 and FEP2 benchmark can be downloaded from the corresponding **Refs 26 and 30**. The targets in FEP2 are more challenging than those in the FEP1 dataset, where ligand perturbation includes changes in the net charge and charge distribution of the molecule, in addition to ring breakage and scaffold hopping.

BindingNet-AC benchmark. This study further assessed model generalization and potential in OOD/real drug discovery scenarios against other data-driven models using the BindingNet-AC benchmark published by Li *et al.*[31] The initial structures of this dataset are available from the BindingNet website (http://bindingnet.huanglab.org.cn/). We only used

cases with crystal structure templates, resulting in 51,131 ligand pairs across 455 targets and 25,938 ligands. The selection criteria for AC pairs and the protocols for 3D structure modeling follow those reported in **Ref 31**.

DeepBAR benchmark. This study utilized the CB7 host and four guests studied in DeepBAR, as reported by Ding et al.[32], to assess the efficacy of LamNet for ABFE prediction preliminarily. The preliminary structural preparation involved (1) downloading the host structure from the SAMPL4 GitHub URL (https://github.com/samplchallenges/SAMPL4), (2) converting the guest SMILES into a 3D structure utilizing RDKit, and (3) manually positioning the guests within the host pocket using Maestro (Schrödinger Inc.).

Performance indicators

The RMSE and Pearson r metrics were used to evaluate the prediction performance of LamNet, where RMSE provides the accuracy between LamNet-calculated values and experimental values:

$$\mathrm{RMSE} = \sqrt{\frac{1}{|\mathcal{D}|}\sum_{n=1}^{|\mathcal{D}|}(\widehat{y_n} - y_n)^2} \#(37)$$

where $\mathcal{D}$ represents the dataset, $\widehat{y_n}$ represents the calculated values, and y_n represents the experimental values. Pearson r provides the correlation between LamNet prediction values and the experimental values, reflecting the ranking performance of LamNet:

$$R_p = \frac{\sum_{i=1}^{|\mathcal{D}|}(\hat{y}i - \bar{\hat{y}})(y_i - \bar{y})}{\sqrt{\sum_{i=1}^{|\mathcal{D}|}(\hat{y}i - \bar{\hat{y}})^2}\sqrt{\sum_{i=1}^{|\mathcal{D}|}(y_i - \bar{y})^2}} \#(38)$$

where $\bar{\hat{y}}$ and $\bar{y}$ represent the means of the predicted and experimental values, respectively.

LamNet and its variants

- **LamNet_w** (default): The default model setting used in the main text. The subscript "w" denotes the loss weighting strategy, where data points are reweighted during training to prioritize of high-quality samples and reduce the influence of low-quality or noisy ones.
- **LamNet**: An ablation variant in which the loss weighting strategy is removed, such that all

data points are treated with equal weight during training.

- **LamNet_p**: An ablation variant that additionally introduces input structure preprocessing. On top of the unweighted model, two types of preprocessing are applied: (1) alignment of ligands based on their maximum common substructure (MCS), and (2) preprocessing of the whole system by minimization and equilibration before extracting model inputs.
- **LamNet_p_w**: A variant that extends the default weighted model (LamNet_w) by adding the same input structure preprocessing steps as described above, thereby combining reweighting and preprocessing.

Data-driven models in comparison

- **PBCNet**[23]: Constructs a joint protein–ligand interaction graph and employs alternating message passing and dual-task learning to predict relative binding affinities. The model was trained on ~600k ligand pairs derived from the PDBBind database.
- **PBCNet2**[1]: Extends PBCNet with a Cartesian tensor message passing mechanism for fine-grained spatial representation, improving prediction accuracy and generalization. The training data include additional docking-based conformational ensembles.
- **PIGNet2**[33]: Incorporates physics-inspired inductive biases and data augmentation to improve affinity prediction and generalization. We used the pda_0 checkpoint with default settings.
- **OnionNet2**[34]: A CNN-based model that predicts binding affinity by modeling the distribution of contact points across multi-shell distance layers.
- **RTMScore**[35]: Scores and ranks ligand binding poses by fitting distance likelihood potentials via a mixture density network.
- **GenScore**[36]: Extends RTMScore with a multi-objective framework balancing docking, ranking, and scoring tasks.
- **Boltz2**[37]: Combines co-folding–based structural representation learning with reverse diffusion–based structure optimization, followed by a prediction head for affinity estimation.

Relative binding free energy prediction: ablation experiments and factor analysis

To further understand specific contributions of design choices and the impact of data and input structure preprocessing on LamNet, we performed ablation experiments and factor analysis across various RBFE prediction cases, concentrating on the following main aspects: (1) loss function design, (2) perturbation graph connectivity, and (3) structural input preprocessing. The results are summarized in **Figure S4**.

Initially, we analyzed the impact of a loss weighting strategy that emphasizes high-reliability data during training. The RBFE prediction procedures for each target are the same as those described in the main body. Various test sets associated with the same target can also be distinguished by numbers of ligands in parenthesis. The only difference is that in this paragraph, we use the terms LamNet and LamNet_w to distinguish whether the loss is weighted. **Figure 5a** illustrates that LamNet_w, utilizing the accuracy-based weighting, consistently surpasses its unweighted counterpart across most evaluated systems. This demonstrates that the strategy of improving the overall performance of LamNet via loss weighting by accuracy is effective, thereby affirming that the training data quality significantly impacts LamNet's learning experience. It should be noted that cMet (11) and CDK8 (33) systems exhibit some mild exceptions. The possible reasons are (1) the weighted loss data may lose vital details regarding conformational or physical changes related to the two systems (setting the weights of some important ligand pairs transformation data very small due to the computational errors); (2) the conformational complexity of proteins and ligands in both systems is complicated, and the weighting would result in sparse data and inadequate learning. Future work may gain from adaptive weighting algorithms that account for both error magnitude and chemical diversity, or from the dynamic re-weighting of data according to local learning gradients.

Secondly, we investigated the impact of the ligand mapping strategy, particularly how it differentiates between strongly connected and weakly connected, on LamNet's performance. As shown in **Figure S4b**, adopting weak connectivity enhances Pearson r for the majority of the 16 systems. This suggests that the model exhibits greater susceptibility to large structural perturbations, hence supporting the claim that the framework, embedding both local and global molecular contexts through data-driven representation learning, enables LamNet to learn flexible correspondences between perturbed substructures and avoids the explicit sampling bottleneck. In contrast, conventional AFEMs must strictly scale the interaction terms associated

with the perturbed structures and rely on explicit dynamical sampling. When faced with large perturbations, this leads to complex energy term handling and rough configurational spaces, often resulting in slow convergence and large errors[26,30]. As a result, this proves that LamNet can serve as a formidable tool at the LO stage to handle scenarios that were previously avoided due to the lack of reliable computational methods.

At last, we evaluated the effects of the input preprocessing, wherein ligand alignment was executed for the cMet system, and system minimization and pre-equilibration were conducted for the thrombin and pfkfb3 systems. In this paragraph, we use the terms LamNet_w and LamNet_p_w to clearly indicate whether the preprocessing is used. **Figure S4c** shows that aligning ligands based on their maximum common substructure (MCS) is detrimental to the performance of LamNet. Preprocessing structures for the whole system via minimization and quick equilibration are favorable for LamNet. This phenomenon may be explained by the fact that aligning the ligand substructures would not only deviate from the actual conformational diversity but also introduce artificial rigidity to the system. Preprocessing the conformation of the entire system will yield a more realistic and precise initial conformation. This additionally suggests that LamNet's robustness to conformational perturbations is not as strong as that of traditional MD-based ensemble approaches. Future effort may benefit either from incorporating a greater diversity of MD-sampled conformations into the training dataset or by introducing explicit structural noise/perturbations during training.

These investigations demonstrate that LamNet's efficacy is closely associated with the quality of training data, the mapping scheme used, and the uniformity of accurate inputs. The current framework provides reliable and generalizable RBFE predictions; however, these results reveal substantial opportunity for further improvement via adaptive loss functions, more chemically informed mapping strategies, standardized accurate structure preprocessing, and dataset construction or model design that take conformational diversity into account. These discoveries prove LamNet's potential for growth into a physically grounded and data-efficient framework capable of tackling complicated, large-scale, and low-data drug discovery situations with unprecedented speed and accuracy.

LamNet framework: feasibility analysis when integrating with other AFEMs

To assist researchers interested in extending LamNet or developing new models based on this paradigm, we provide here a practical discussion on the feasibility of integrating LamNet with other AFEMs, covering both data preparation and model adaptation aspects.

Computational cost of dataset generation. Using RBFE as an example, the relative computational cost of different AFEMs varies considerably. If the cost of generating an ATM dataset is defined as 1×, standard FEP or TI simulations typically require about 2× due to their more complex simulation protocols. The actual cost is also influenced by the desired data quality — higher-accuracy datasets demand longer simulation times. Based on our experience, a fully converged and reliable FEP calculation for a single ligand-pair typically takes about one month on a single GPU. While enhanced sampling techniques can reduce this cost, it remains substantial. Targeted sampling of windows that cross deep potential wells or high-energy barriers is another effective strategy to improve data quality without prohibitively increasing cost. Notably, LamNet itself could be leveraged to accelerate dataset generation by optimizing window settings. Constructing high-quality ABFE datasets is inherently more expensive, requiring more stringent sampling and longer simulation times. Nevertheless, the general strategies outlined above remain applicable.

Theoretical considerations for model adaptation. Figure S5 schematically compares the workflows of ATM and classical AFEMs such as FEP and TI (represented by the double decoupling method, DDM). The DDM framework involves more complex treatments of various restraints and interaction terms, as well as a longer, multi-stage transformation pathway to reach intermediate states. If LamNet is to be adapted to DDM, these factors must be carefully incorporated into the model design and training objectives.

It is worth noting that methods like λ-dynamics do not require predefined intermediate windows, and their computational cost is only ~0.1–0.01× of ATM. However, defining physical processes and labels for such methods is considerably more challenging. Moreover, they are typically applicable only to RBFE calculations. For these reasons, we did not include a detailed discussion of LamNet's potential integration with λ-dynamics methods here.

References

1. Yu J, Sheng X, Fan Z et al. 1 advancing ligand binding affinity prediction with cartesian n.d.
2. Sabanés Zariquiey F, Pérez A, Majewski M et al. Validation of the Alchemical Transfer Method for the Estimation of Relative Binding Affinities of Molecular Series. *J Chem Inf Model*. 2023; 63: 2438–44.
3. Maier JA, Martinez C, Kasavajhala K et al. ff14SB: Improving the accuracy of protein side chain and backbone parameters from ff99SB. *J Chem Theory Comput*. 2015; 11: 3696–713.
4. He X, Liu S, Lee T-S et al. Fast, Accurate, and Reliable Protocols for Routine Calculations of Protein–Ligand Binding Affinities in Drug Design Projects Using AMBER GPU-TI with ff14SB/GAFF. *ACS Omega*. 2020; 5: 4611–9.
5. Sabanés Zariquiey F, Galvelis R, Gallicchio E et al. Enhancing protein–ligand binding affinity predictions using neural network potentials. *J Chem Inf Model*. 2024; 64: 1481–5.
6. Galvelis R, Varela-Rial A, Doerr S et al. NNP/MM: Accelerating Molecular Dynamics Simulations with Machine Learning Potentials and Molecular Mechanics. *J Chem Inf Model*. 2023; 63: 5701–8.
7. Eastman P, Swails J, Chodera JD et al. OpenMM 7: Rapid development of high performance algorithms for molecular dynamics. *PLOS Comput Biol*. 2017; 13: e1005659.
8. Wu JZ, Azimi S, Khuttan S et al. Alchemical transfer approach to absolute binding free energy estimation. *J Chem Theory Comput*. 2021; 17: 3309–19.
9. O'Boyle NM, Banck M, James CA et al. Open Babel: An open chemical toolbox. *J Cheminformatics*. 2011; 3: 33.
10. Cock PJA, Antao T, Chang JT et al. Biopython: freely available Python tools for computational molecular biology and bioinformatics. *Bioinformatics*. 2009; 25: 1422–3.
11. Schrödinger L. The PyMOL Molecular Graphics System, Version 2.0 2015.
12. Landrum G. RDKit documentation n.d.
13. Muddana HS, Fenley AT, Mobley DL et al. The SAMPL4 host–guest blind prediction challenge: an overview. *J Comput Aided Mol Des*. 2014; 28: 305–17.
14. Yin J, Henriksen NM, Slochower DR et al. Overview of the SAMPL5 host–guest challenge: Are we doing better? *J Comput Aided Mol Des*. 2017; 31: 1–19.

15. Rizzi A, Murkli S, McNeill JN et al. Overview of the SAMPL6 host–guest binding affinity prediction challenge. *J Comput Aided Mol Des*. 2018; 32: 937–63.

16. Amezcua M, El Khoury L, Mobley DL. SAMPL7 Host–Guest Challenge Overview: assessing the reliability of polarizable and non-polarizable methods for binding free energy calculations. *J Comput Aided Mol Des*. 2021; 35: 1–35.

17. Amezcua M, Setiadi J, Ge Y et al. An overview of the SAMPL8 host–guest binding challenge. *J Comput Aided Mol Des*. 2022; 36: 707–34.

18. Khuttan S, Azimi S, Z. Wu J et al. Taming multiple binding poses in alchemical binding free energy prediction: The β-cyclodextrin host–guest SAMPL9 blinded challenge. *Phys Chem Chem Phys*. 2023; 25: 24364–76.

19. Trott O, Olson AJ. AutoDock vina: Improving the speed and accuracy of docking with a new scoring function, efficient optimization, and multithreading. *J Comput Chem*. 2010; 31: 455–61.

20. Case DA, Aktulga HM, Belfon K et al. Amber 2023. *University of California, San Francisco* 2023; .

21. Doerr S, Harvey MJ, Noé F et al. HTMD: High-throughput molecular dynamics for molecular discovery. *J Chem Theory Comput*. 2016; 12: 1845–52.

22. Yang Z, Zhong W, Lv Q et al. Geometric Interaction Graph Neural Network for Predicting Protein–Ligand Binding Affinities from 3D Structures (GIGN). *J Phys Chem Lett*. 2023; 14: 2020–33.

23. Yu J, Li Z, Chen G et al. Computing the relative binding affinity of ligands based on a pairwise binding comparison network. *Nat Comput Sci*. 2023.

24. Loshchilov I, Hutter F. Decoupled weight decay regularization 2019.

25. Paszke A, Gross S, Massa F et al. PyTorch: An imperative style, high-performance deep learning library. Adv. Neural Inf. Process. Syst., vol. 32; *Curran Associates, Inc.* 2019; .

26. Wang L, Wu Y, Deng Y et al. Accurate and Reliable Prediction of Relative Ligand Binding Potency in Prospective Drug Discovery by Way of a Modern Free-Energy Calculation Protocol and Force Field. *J Am Chem Soc*. 2015; 137: 2695–703.

27. Azimi S, Wu JZ, Khuttan S et al. Application of the alchemical transfer and potential of mean force methods to the SAMPL8 host-guest blinded challenge. *J Comput Aided Mol Des*.

2022; 36: 63–76.

28. Zeng J, Qian Y. Adaptive lambda schemes for efficient relative binding free energy calculation. *J Comput Chem*. n.d.; n/a.

29. Robo MT, Hayes RL, Ding X et al. Fast free energy estimates from λ-dynamics with bias-updated Gibbs sampling. *Nat Commun*. 2023; 14: 8515.

30. Schindler CEM, Baumann H, Blum A et al. Large-Scale Assessment of Binding Free Energy Calculations in Active Drug Discovery Projects. *J Chem Inf Model*. 2020; 60: 5457–74.

31. Li X, Shen C, Zhu H et al. A High-Quality Data Set of Protein-Ligand Binding Interactions Via Comparative Complex Structure Modeling. *J Chem Inf Model*. 2024; 64: 2454–66.

32. Ding X, Zhang B. DeepBAR: A Fast and Exact Method for Binding Free Energy Computation. *J Phys Chem Lett*. 2021; 12: 2509–15.

33. Moon S, Hwang S-Y, Lim J et al. PIGNet2: A versatile deep learning-based protein–ligand interaction prediction model for binding affinity scoring and virtual screening. *Digit Discov*. 2024; 3: 287–99.

34. Wang Z, Zheng L, Liu Y et al. OnionNet-2: A convolutional neural network model for predicting protein-ligand binding affinity based on residue-atom contacting shells. *Front Chem*. 2021; 9: 753002.

35. Shen C, Zhang X, Deng Y et al. Boosting protein–ligand binding pose prediction and virtual screening based on residue–atom distance likelihood potential and graph transformer. *J Med Chem*. 2022; 65: 10691–706.

36. Shen C, Zhang X, Hsieh C-Y et al. A generalized protein–ligand scoring framework with balanced scoring, docking, ranking and screening powers. *Chem Sci*. 2023; 14: 8129–46.

37. Boltz-2: Towards Accurate and Efficient Binding Affinity Prediction n.d.
